# The Complexity of Posttranscriptional Small RNA Regulatory Networks Revealed by *In Silico* Analysis of *Gossypium arboreum* L. Leaf, Flower and Boll Small Regulatory RNAs

**DOI:** 10.1371/journal.pone.0127468

**Published:** 2015-06-12

**Authors:** Hongtao Hu, Aaron M. Rashotte, Narendra K. Singh, David B. Weaver, Leslie R. Goertzen, Shree R. Singh, Robert D. Locy

**Affiliations:** 1 Department of Biological Sciences, Auburn University, Auburn, AL 36849, United States of America; 2 Department of Crop, Soil & Environmental Sciences, Auburn University, Auburn, AL 36849, United States of America; 3 Center for Nano Biotechnology Research, Alabama State University, Montgomery, AL 33104, United States of America; National Key Laboratory of Crop Genetic Improvement, CHINA

## Abstract

MicroRNAs (miRNAs) and secondary small interfering RNAs (principally phased siRNAs or trans-acting siRNAs) are two distinct subfamilies of small RNAs (sRNAs) that are emerging as key regulators of posttranscriptional gene expression in plants. Both miRNAs and secondary-siRNAs (sec-siRNAs) are processed from longer RNA precursors by DICER-LIKE proteins (DCLs). *Gossypium arboreum* L., also known as tree cotton or Asian cotton, is a diploid, possibly ancestral relative of tetraploid *Gossypium hirsutum* L., the predominant type of commercially grown cotton worldwide known as upland cotton. To understand the biological significance of these gene regulators in *G*. *arboreum*, a bioinformatics analysis was performed on *G*. *arboreum* small RNAs produced from *G*. *arboreum* leaf, flower, and boll tissues. Consequently, 263 miRNAs derived from 353 precursors, including 155 conserved miRNAs (cs-miRNAs) and 108 novel lineage-specific miRNAs (ls-miRNAs). Along with miRNAs, 2,033 miRNA variants (isomiRNAs) were identified as well. Those isomiRNAs with variation at the 3’-miRNA end were expressed at the highest levels, compared to other types of variants. In addition, 755 pha-siRNAs derived 319 pha-siRNA gene transcripts (PGTs) were identified, and the potential pha-siRNA initiators were predicted. Also, 2,251 non-phased siRNAs were found as well, of which 1,088 appeared to be produced by so-called *cis*- or *trans*-cleavage of the PGTs observed at positions differing from pha-siRNAs. Of those sRNAs, 148 miRNAs/isomiRNAs and 274 phased/non-phased siRNAs were differentially expressed in one or more pairs of tissues examined. Target analysis revealed that target genes for both miRNAs and pha-siRNAs are involved a broad range of metabolic and enzymatic activities. We demonstrate that secondary siRNA production could result from initial cleavage of precursors by both miRNAs or isomiRNAs, and that subsequently produced phased and unphased siRNAs could result that also serve as triggers of a second round of both *cis*- and *trans*-cleavage of additional siRNAs, leading to the formation of complex sRNA regulatory networks mediating posttranscriptional gene silencing. Results from this study extended our knowledge on *G*. *arboreum* sRNAs and their biological importance, which would facilitate future studies on regulatory mechanism of tissue development in cotton and other plant species.

## Introduction

Small regulatory RNAs (sRNAs), have been found in a wide variety of plants from mosses to eudicots [1–8] and mediate a broad range of biological processes, including growth, development, and stress responses [1,2,9–11]. Based on origin and biogenesis, these gene regulators can be divided into at least two major classes [12], microRNAs (miRNAs) and small interfering RNAs (siRNAs), each of which can be further divided into subsequent groups.

In plants, miRNAs appear to be processed from single-stranded precursors that are transcribed by RNA polymerase II (*Pol*II), which are capable of self-folding into hair-pin shaped secondary structures (known as hairpins or stem-loops) and are subsequently processed by a specific set of nucleases referred to as DICER-LIKE proteins (DCLs) [13,14] with assistance of DRB (Double-stranded RNA Binding) proteins [15,16]. Canonical miRNAs, typically ~21 nt or 22 nt in length, are processed by the DCL1/AGO1 pathway [13,14] and apparently mediate gene expression posttranscriptionally by either cleavage of or translation suppression of target mRNAs, respectively [17,18]. Other long miRNAs (23- or 24-nt) are produced by the /DCL3/AGO4 pathway and appear to regulate DNA methylation [19,20] and transcriptional changes in gene expression.

In contrast to miRNAs, small interfering RNAs (siRNAs) are derived from various non-hairpin RNA double-stranded RNA precursors [21]. In plants, siRNAs include secondary siRNAs that can be either phased siRNAs (pha-siRNAs), also known as *trans*-acting siRNAs (ta-siRNAs); or non-phased siRNAs based on linear sequential cleavage or apparently non-sequential cleavage, respectively. Other types of siRNA include heterochromatic siRNAs (hc-siRNAs), sometimes referred to as repeat-associated siRNAs (ra-siRNAs) that are typically 24 nt in length [22,23] and nat-siRNAs are produced from a pair of natural antisense transcripts (NAT pairs) that are independently transcribed from sense and antisense strands that are either produced from NAT pairs derived from the same genomic locus (*cis*-nat-siRNAs) or derived from NAT pairs transcribed from distinct genomic loci (*trans*-nat-siRNAs) [8,24]. However, the mechanism by which these classes of small RNA mediate gene expression is less well understood at this time although their length distribution suggests that they most likely work other than posttranscriptionally.

Secondary siRNAs are sRNAs produced from phased siRNA gene transcripts (PGTs) following cleavage of a primary transcript by miRNAs or other siRNAs [6,25]. The biogenesis of pha-siRNAs requires the activities of RDR6/SGS3/DCL4 [26–28]. Typically, pha-siRNAs are 21 or 22 nt in length, but 24 nt long pha-siRNAs have also recently been found in plants [29,30]. It has been shown that miRNA-directed pha-siRNA pathways play crucial roles in plant developmental timing [4] and disease resistance [9].

To date, two different models of the biogenesis of pha-siRNAs are proposed, including so-called “one-hit” and “two-hit” model [5,26]. In the “one-hit” model, PGTs are initially cleaved by one miRNA that generates only one cleavage site up-stream of siRNA transcripts, whereas in the “two-hit” model, both an up- and down-stream target sites are located within the PGT [4,5]. Known pha-siRNA-yielding genes such as NBS-LRRs (Nucleotide-Binding Site Leucine-Rich Repeat) and ARFs (Auxin Response Factors), along with their master miRNAs (miR382 and miR390), are found in various plants, including *Physcomitrella*, *Medicago*, *Arabidopsis*, and *Vitis* [9,31–33], suggesting that at least these pha-siRNA regulatory pathways are evolutionarily conserved in plants.

Cotton (*Gossypium spp*.) is an economically important fiber crop worldwide. Diploid *Gossypium arboreum* L. (also known as: tree, perennial, or Asian cotton) possesses many favorable traits for cotton production, which the more commercially exploited tetraploid, upland cotton (*Gossypium hirsutum* L.) cultivars lack, such as drought tolerance and resistance to diseases or insects [34,35].

Although miRNAs are documented in tetraploid upland cotton [36–38], much less is known about sRNAs and their roles in diploid *G*. *arboreum*. To better understand the regulatory roles of sRNAs in cotton tissue development, a genome-wide investigation of small regulatory RNAs was performed using three libraries of small RNAs derived from a mixture of leaves, flower and boll, which were sequenced using Illumina deep sequencing technology. Bioinformatic analysis of these libraries yielded a large number of conserved miRNAs (cs-miRNAs) and lineage-specific miRNAs (ls-miRNAs). In addition, a large number of length variants (isomiRNAs) and conserved and novel pha-siRNAs and non-pha-siRNAs were also identified. These can be utilized to formulate putative small RNA regulatory networks associated with a broad spectrum of biological processes and molecular functions associated with tissue development in *G*. *arboreum*.

## Methods

### The pre-assembled genome and EST datasets of *G*. *arboreum*

Whole genome sequencing reads of *Gossypium arboreum* L. were downloaded from the Comparative Evolutionary Genomics of Cotton site (http://128.192.141.98/CottonFiber/), and the genome was preliminarily assembled using CLC Assembly Cell 2.0 (http://www.clcbio.com) using default parameters. This assembly generated 190,728 contigs, ranging from 100 to 64,834 nt in length, with an average contig size of 700 nt. The average coverage and N50 of the assembly was 34.4X and 1,551-nt, respectively.

*G*. *arboreum* ESTs (Expressed Sequence Tags) were collected from both NCBI (http://www.ncbi.nlm.nih.gov/) and Comparative Evolutionary Genomics of Cotton (http://128.192.141.98/CottonFiber/).

The *G*. *arboreum* sequence library (GARSL), constituting of the pre-assembled *G*. *arboreum genome* and all ESTs (also described above), was constructed and used for identification of miRNAs and pha-siRNAs in this study.

### *G*. *arboreum* Small RNA libraries

Three *G*. *arboreum* small RNA sequencing libraries used in this study were downloaded from the Comparative Sequencing of Plant Small RNAs website (http://smallrna.udel.edu), which are also available in NCBI GEO database (http://www.ncbi.nlm.nih.gov/geo/query/acc.cgi?acc=GSE28965). According to information provided by the Comparative Sequencing of Plant Small RNAs website (http://smallrna.udel.edu), the three *G*. *arboreum* sRNA libraries were constructed from the youngest leaf on the top of plants, a mixture of -3 to 0 DPA (days post anthesis) flowers, and 8–10 DPA bolls without ovules. Those three small RNA libraries were sequenced, using Illumina deep sequencing (Illumina Genome Analyzer II). Adapters were trimmed from these sequences, and the three nearly equal sized libraries were merged into a putative total sRNA library containing 20,562,881 reads that were collapsed into 8,668,051 unique sequences (S1 Table).

These reads were filtered for sequences 18 to 26 nt in length in each library and the merged library (19,745,542 reads, 8,167,667 unique sequences), and those reads perfectly mapping to the GARSL (see above) were chosen for further study (S1 Table). After filtration, approximately 5.5 million unique sequences were retained, and all of these sequences were considered as the putative sRNA sequences of *G*. *arboreum* (GarpsRNA library).

### Bioinformatics analysis of sRNA deep sequencing data

The general approach for sRNA analysis, the miRNA analysis pipeline, and the pha-siRNA analysis pipeline are outlined in S1, S2 and S3 Figs, respectively. Individual steps in the processes were performed, using in-house Perl scripts combined with miREAP (http://sourceforge.net/projects/mireap), CentroidFold [39], The UEA small RNA Workbench [40], and psRNATarget [41].

In order to identify miRNAs, a bioinformatics pipeline was developed as described in S2 Fig. This pipeline was designed to discover miRNAs using the plant miRNAs features described in previous studies [42–44].

First, sRNAs with a total read abundance of 10 or more were submitted to a modified version of miREAP (http://sourceforge.net/projects/mireap/), in which a shift of up to three nucleotide from putative miRNA loci was allowed, for extraction of miRNA precursors, using the maximal distance between miRNA and miRNA* (450-nt), the maximal free energy of -30 kcal mol^-1^, the maximal loci of 500 and defaults for others. The remaining putative miRNAs were filtered by two extra features of plant miRNAs, single strand bias (≥0.9, the total reads of sRNAs from the sense strand of miRNA precursor divided by the total reads of sRNA from both strands of miRNA precursors) and an abundance bias (≥0.6, the total reads of the most abundant three sRNAs derived from putative miRNA loci divided by the total reads of sRNAs matching the miRNA precursors) [9,43]. Last, the secondary structures of putative miRNA precursors were evaluated, using CentroidFold [39]. Those miRNA precursors in which four or less mismatches were found between miRNA and miRNA* and that had appropriate parameters including, minimum free energy index (0.30 to 1.80), adjusted minimum free energy (≥22 kcal/mol/100nt), GC content (≥25%), were selected [44]. miRNAs corresponding to these criteria were considered as miRNA candidates. Sequences derived from miRNA loci but that failed to be annotated as miRNAs were considered as miRNA variants (also known as isomiRNAs).

sRNAs in the GarpsRNA library with a total abundance of ≥ 2 reads that were not derived from miRNA loci were used as the input of the pha-siRNA analysis pipeline (S3 Fig).

The putative pha-siRNA loci were identified using The UEA Small RNA Workbench [40] with a p-value cutoff of < 0.01. The putative pha-siRNA loci were further filtered by three characteristics: 1) a pha-siRNA with a total read abundance of 10 or more and was produced from a putative pha-siRNA locus; 2) the total abundance of pha-siRNAs was equal to or greater than 50% of the total reads from each precursor; and 3) the strand bias was smaller than 0.9. The triggers of pha-siRNA gene transcripts (PGTs) were predicted by a combination of an in-house Perl script and psRNATarget [41], following methodology described in previous studies [30,45]. Target analysis of pha-siRNAs were conducted using psRNATarget [41], against the *G*. *arboreum* ESTs collection described above.

### Analysis of gene ontology of miRNA and pha-siRNA targets

The annotation of miRNA targets or PGTs was performed by BLASTX or BLASTN searching against UniProt Knowledgebase (http://www.uniprot.org) [46,47], and/or NCBI non-redundant protein database (http://www.ncbi.nih.gov), with default parameters. The analysis of gene ontology was completed, using Blast2GO [48,49], or AgriGO [50].

### Statistical analysis

Expression analysis of miRNAs, isomiRNAs, pha-siRNAs and non-pha-siRNAs in different tissues was conducted using Kal’s test as implemented in CLC Genome WorkBench (version 5.8, http://www.clcbio.com). To perform the statistical test, the reads of miRNAs or pha-siRNAs in each sRNA library were normalized into reads per million reads (RPM). The FDR corrected p-value < 0.05 was used as the cutoffs to indicate a significant difference by multiple comparisons (leaf vs. flower, leaf vs. boll, or flower vs. boll).

The statistical analysis of Least Significant Difference (LSD) was performed, using IBM SPSS (http://www-01.ibm.com/software/analytics/spss/).

### Hierarchical cluster analysis

The hierarchical cluster analysis for expression of sRNAs among different tissues was performed, using R Open Source software (version 3.03, http://www.r-project.org). The sRNAs that were differentially expressed between at least one pair of tissues were used for the clustering. The normalized Z-score expression value $\left(\text{Z score}=\frac{\text{Expression value}-\text{Mean}}{\text{Standard deviation}}\right)$ was calculated and used for this analysis.

## Results and Discussion

### Deep sequencing of *G*. *arboreum* small RNA libraries

**Fig 1:**
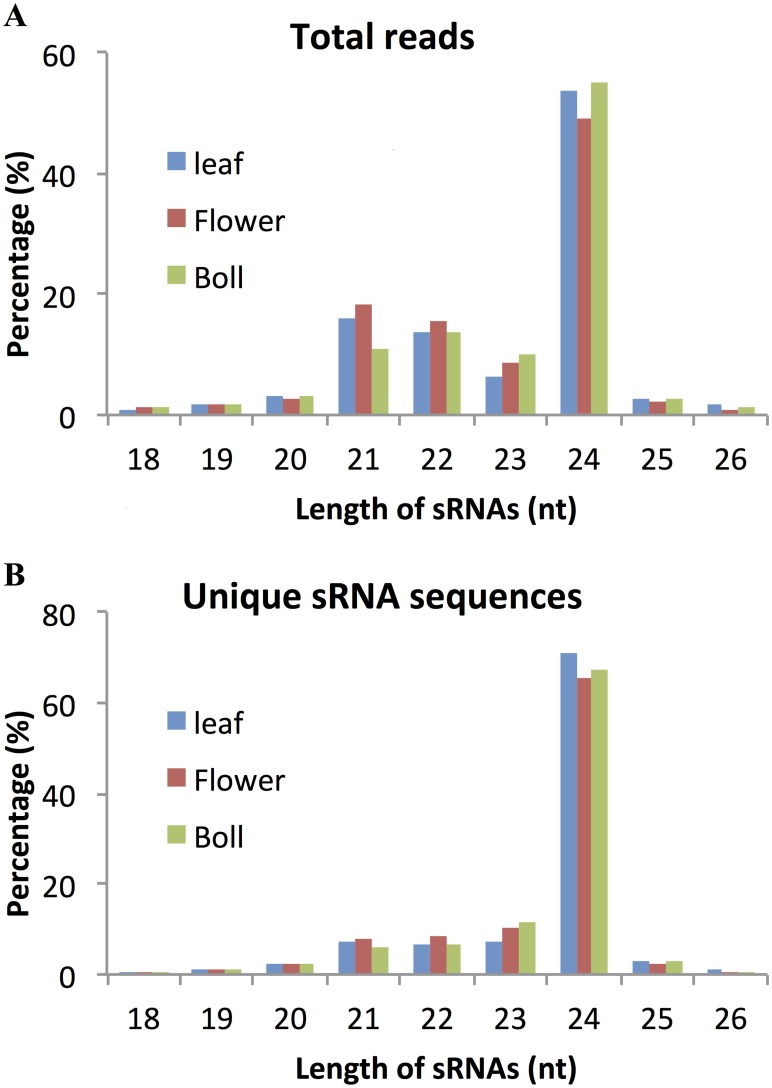
Size distribution of small RNAs from *G. arboreum* leaf, flower, and boll tissues. Size distribution of the total libraries from the leaf, flower, and boll tissues are shown as indicated. The percentage of total reads versus read length is shown in panel A, and the percentage of unique sequences compared to the total number of unique sequences is shown for each size class in panel B.

Three small RNA libraries constructed and sequenced from *G*. *arboreum* leaf, flower and boll were obtained from the Comparative Sequencing of Plant Small RNAs website (http://smallrna.udel.edu). Summary statistics for the adapter-trimmed and GARSL-matching sequences are given in S1 Table. Note that in each library approximately 75% of the total reads qualified as matching to the *G. arboreum* genome, suggesting that the libraries were of relatively high quality. These sequence reads were subjected to small RNA analysis (see S1 Fig). sRNA sequences from each tissue library that exactly matched the GARSL shared a similar length distribution pattern (Fig 1). The sRNA sequences 24 nt in length were the most abundant in all three tissues examined, corresponding to 49–55% of total reads (Fig 1, panel A) and 66–71% of the unique putative sRNA sequences (Fig 1, panel B) in each library. The sRNA sequences 21 and 22 nt in length each represented 11–18% of the total reads in each library, but reflected slightly less than 10% of the unique putative sRNA sequences. This reflects what appears to be higher average expression percentage for the 21 and 22 nt length classes, and may be related to the function of sRNAs of these lengths. Total reads of sRNAs 23 nt in length varied from 7–10%, and unique putative sRNA sequences of the same length varied from 7–12%. In plants, siRNAs 24 nt in length have been shown to play a role in epigenetic regulation [51,52], and the highly expressed sequences 24 nt in length in both vegetative and reproductive tissues found here, further supports the notion that they play key roles in tissue-specific and developmental regulation.

### Identification of miRNAs and their variants in *G*. *arboreum*

Unique putative sRNA sequences with read counts greater than 10 in at least one library and that perfectly matched the GARSL were subjected to further analysis using the bioinformatics pipeline described in S2 Fig. Those sequences, which satisfied the criteria of plant miRNAs as described in previous studies [9,42–44], were annotated as putative miRNAs. A total of 353 putative miRNA hairpin precursors were identified in the *G*. *arboreum* genome and EST assemblies (S2 Table). These precursors produced a total of 263 unique putative mature miRNAs (Table 1). Of these putative mature miRNA sequences, 155 sRNAs derived from 236 precursor hairpins matched known plant miRNAs registered in miRBase (Release 21, http://www.mirbase.org/) with no more than three base mismatches. These were considered putative conserved miRNAs (cs-miRNAs) and annotated according to the corresponding miRBase21 family to which they belonged (S2 Table). A few miRNAs with their star sequences (miRNA*, the complementary pattern sequence of a miRNAs) being highly similar to known plant miRNAs in sequence, were also considered as cs-miRNA candidates [42]. Of the cs-miRNA, many (131/155 mature cs-miRNAs potentially derived from 202/236 cs-miRNA precursors) were previously found in the genus *Gossypium* as reported in miRBase 21. These included 82 of the 296 miRNAs reported for the *Gosspyium raimondii* Ulbr. genome in miRBase21 [53,54], and 46 of the 80 *G*. *hirsutum* miRNAs reported in miRBase21.

**Table 1:** Summary for *G. arboreum* miRNAs.

	cs-miRNAs		ls-miRNAs	Total
**A.**	**Predicted hairpin precursors**	236	117	353
	**miRBase21:**			
	*** Gossypium***	202		
	*** G*.*raimondii***	112		
	*** G*. *herbaceum***	6		
	*** G*. *arboreum***	1		
	** G.hirsutum**	83		
	** Other Viridiplantae**	34		
**B.**	**Predicted mature miRNAs**	155	108	263
	**miRBase21:**			
	*** Gossypium***	131		
	*** G*.*raimondii***	82		
	*** G*. *herbaceum***	2		
	*** G*. *arboreum***	1		
	** G.hirsutum**	46		
	** Other Viridiplantae**	24		

The number of miRNA precursor sequences producing both cs-miRNAs and and ls-miRNAs are shown in part A. Part B. shows the number of mature cs-miNA and ls-miRNA sequences predicted to be derived from those precursors. In both parts of the table, the number of cs-miRNA sequences obtained from various species in *Gossypium* are shown, and the nmber of sequences obtained from other Viridiplantae sequences are indicated.

One hundred eight putative mature miRNAs found to be different from known plant miRNAs (found in miRBase, Release 21) were annotated as putative novel lineage-specific miRNAs (ls-miRNAs) [12] as they have only been observed in *G*. *arboreum* to date. Sequences shorter than 23 nt in length were considered canonical miRNAs. This included 116/155 cs-miRNAs and 41/108 ls-miRNAs. All remaining sequences were 23 or 24 nt in length and considered noncanonical or long miRNAs [12].

**Fig 2:**
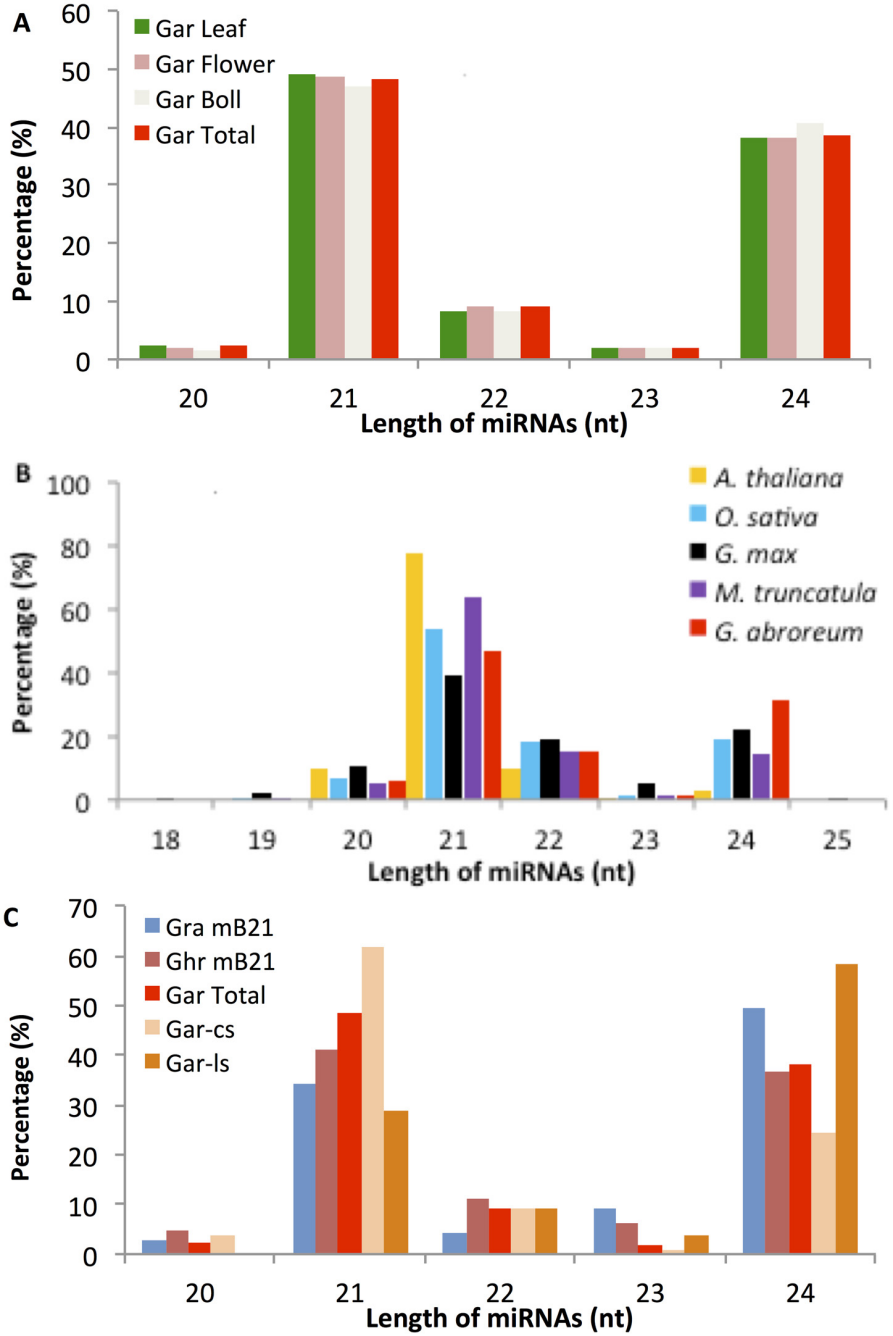
Length Distribution of *G*. *arboreum* predicted miRNAs compared to length distributions of other species species. Panel A. Comparison of the length distribution of mature miRNAs from each tissue-specific library with the *G*. *arboreum* total miRNAs. Panel B. The length distribution of miRNAs derived from *G*. *arboreum* compared to four representative model plant species, *A*. *thaliana*, *Orysa*. *sativa* (rice), *Glycine max*, *Medicago truncatula (barrel medic*) obtained from miRBase v21 are shown. Panel C. Comparison of the length distribution of *G*. *arboreum* total RNA or conserved or lineage specific *G*. *arboreum* miRNAs with the miRNAs found in miRBase v 21 from the genus *Gossypium*, in cluding *G*. *raimondii* (Gra) and G. *hirsutum* (Ghr).

As shown in Fig 2, miRNAs 21 nt in length were predominant and accounted for an average of 48% of *G*. *arboreum* miRNAs in each library and in total (Fig 2, panel A) while miRNAs 24 nt in length were the second most abundant size class, accounting for circa 38% of the miRNAs. Among the cs-miRNAs 62% and 55% were 21 and 24 nt in length respectively, and among the miRNAs deposited in miRBase21 from the genus, *Gossypium*, 41% and 34% of the miRNAs from *G*. *hirsutum* and *G*. *raimondii* respectively were 21 nt in length, while 35% and 50% respectively were found to be 24 nt in length (Fig 2, panel C). Compared to the total Viridiplantae miRBase21 miRNA collection which has but 879/7147 (12.3%) of mature miRNAs that are 24 nt in length, plants with completely sequenced genomes show a more variable percentage of miRNAs 21 nt in length and a consistently lower percentage of miRNAs 24 nt in length. It is, however, not clear whether this represents a true biological phenomenon, or difficulty in deciding the nature and role of 24 nt small RNAs and what can be registered in miRBase.

In plants with completely sequence genomes, it has been suggested that noncanonical miRNAs 24 nt in length play a role in epigenetic regulation through heterochromatic methylation [19], and non-canonical miRNAs 24 nt in length vary from 0.9–49% of total miRNAs found in a Viridiplantae species (miRBase release 21, http://www.mirbas.org) [42,55,56]). The percentage of heterochromatic DNA in the genomes of model species such as *Arabidopsis*, *Oryza*, *Glycine* and *Medicago* roughly correlates with the percentage of miRNAs 24 nt in length [57–60]. Thus, the high percentage of *G*. *arboreum* miRNAs 24 nt in length found here (32.9%) is also consistent with the observations in both other *Gossypium* species and in model species, as *G*. *arboretum* is known to have a high percentage of heterochromatic DNA [61,62]. Additionally, 92% of plant miRNA families 24 nt in length registered in miRBase21 that were lineage-specific, reflecting that these sequences are evolutionarily more recent, and thus they likely have species-specific unique roles in plant developmental processes [63]. In this regard, 63/108 (58%) of the putative unique ls-miRNAs found in this study were 24 nt in length, while only 38/155 (25%) uniqe cs-miRNAs were 24 nt in length (Fig 2, panel C). Thus, the larger percentage of ls-miRNAs 24 nt in length compared to cs-miRNAs further supports the hypothesis that ls-miRNAs 24 nt in length arise from the most variable regions of the genome and are functionally involved in processes other than posttranscriptional gene regulation [63].

In addition to miRNAs, 2,033 miRNA length variants (referred to as isomiRNAs) were predicted to occur in the *G*. *arboreum* datasets (Table 2, S3 Table). These consisted of 1,480 and 553 isomiRNAs derived from precursors for cs-miRNAs and ls-miRNAs respectively. While this is a significant number of isomiRNAs it should also be pointed out that only 11% and 2% of the observed cs- and ls-isomiRNAs were expressed with abundances equal to or greater than 10 RPM (Table 2) but these isomiRNAs represent over 97% of the observed isomiRNA reads in the libraries. Thus, the low abundance cs- and ls-isomiRNA reads are at best speculative at the present time, while those reads with more abundant read counts clearly are representative of typical miRNAs found in higher plants generally and *Gossypium* in general.

**Table 2:** Length distribution and totals for cs-isomiRNAs and ls-isomiRNAs.

	cs-isomiRNA					ls-isomiRNAs				
	RPM		isomiRNA number		Average (RPM/ isomiR)	RPM		isomiRNA #		Average (RPM/ isomiR)
**All-isomiRNAs**										
len = 17	0.0	0.0%	0	0.0%		0.0	0.0%	0	0.0%	
len = 18	1,921.7	2.4%	145	9.8%	13.3	7.0	0.9%	14	2.5%	0.5
len = 19	9,202.9	11.7%	209	14.1%	44.0	40.8	5.3%	30	5.4%	1.4
len = 20	7,630.9	9.7%		16.6%	31.0	118.6	15.4%	43	7.8%	2.8
len = 21	18,093.8	23.0%	297	20.1%	60.9	163.5	21.2%	106	19.2%	1.5
len = 22	40,163.2	51.0%	202	13.6%	198.8	153.9	19.9%	80	14.5%	1.9
len = 23	1,176.2	1.5%	150	10.1%	7.8	73.7	9.5%	95	17.2%	0.8
len = 24	387.3	0.5%	184	12.4%	2.1	198.4	25.7%	157	28.4%	1.3
len = 25	114.0	0.1%	42	2.8%	2.7	13.4	1.7%	22	4.0%	0.6
len = 26	2.1	0.0%	5	0.3%	0.4	2.5	0.3%	6	1.1%	0.4
len = 27	0.0	0.0%	0	0.0%		0.0	0.0%	0	0.0%	
**SUM**	**78,692.1**	**100%**	**1,480**	**100%**	**53.2**	**771.8**	**100%**	**553**	**100%**	**1.4**
**Expression > = 10 RPM—isomiRNAs**										
len = 17	0.0	0.0%	0	0.0%		0.0	0.0%	0	0.0%	
len = 18	1,721.2	2.2%	13	8.3%	132.4	0.0	0.0%	0	0.0%	
len = 19	8,989.8	11.7%	33	21.2%	272.4	16.8	5.3%	1	8.3%	16.8
len = 20	7,329.4	9.5%	41	26.3%	178.8	84.9	26.9%	2	16.7%	42.5
len = 21	17,734.0	23.0%	30	19.2%	591.1	76.5	24.3%	2	16.7%	38.3
len = 22	39,901.4	51.8%	28	17.9%	1425.1	84.3	26.7%	4	33.3%	21.1
len = 23	1,043.7	1.4%	3	1.9%	347.9	0.0	0.0%	0	0.0%	
len = 24	207.1	0.3%	5	3.2%	41.4	52.9	16.8%	3	25.0%	17.6
len = 25	87.0	0.1%	3	1.9%	29.0	0.0	0.0%	0	0.0%	
len = 26	0.0	0.0%	0	0.0%		0.0	0.0%	0	0.0%	
len = 27	0.0	0.0%	0	0.0%		0.0	0.0%	0	0.0%	
**SUM**	**77,013.6**	**100%**	**156**	**100%**	**493.7**	**315.4**	**100%**	**12**	**100%**	**26.3**
Ratio (>10/all)	97.9%		10.5%		9.3	40.9%		2.2%		18.8

**Fig 3:**
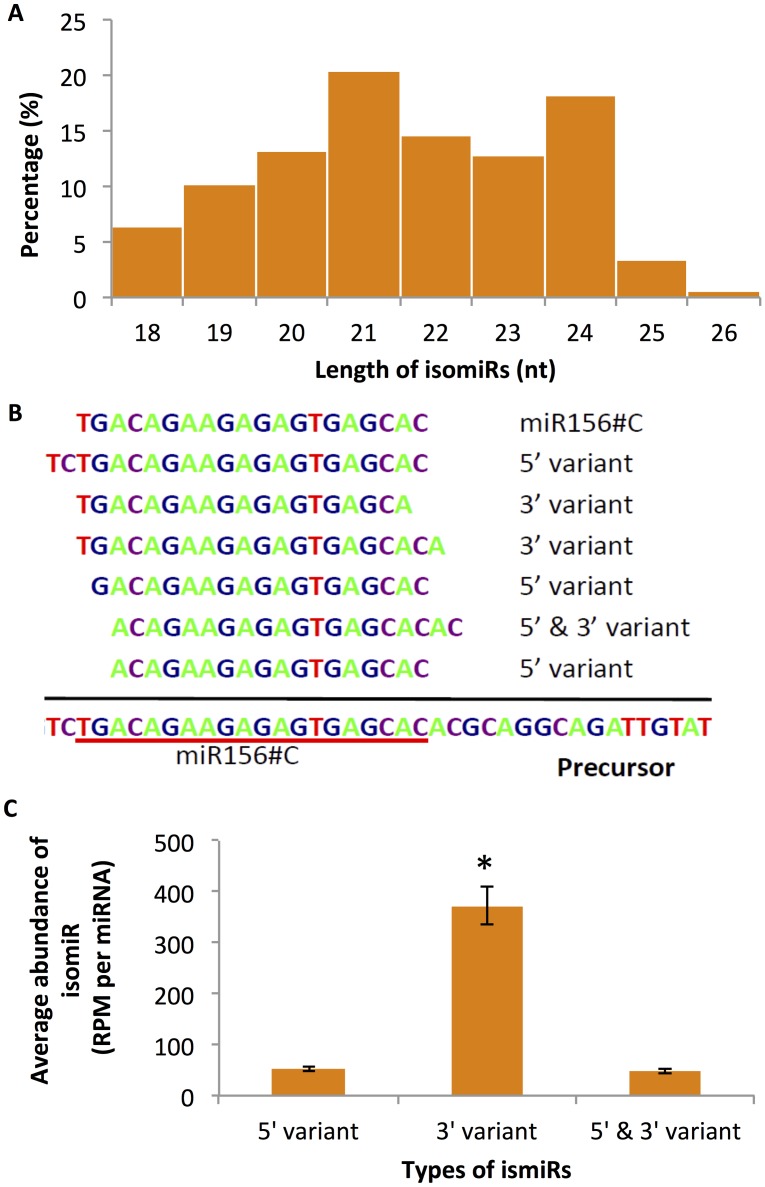
Characteristics of isomiRNAs found in *G*. *arboreum*. The length distribution of all putative miRNA isomiRs is shown in panel A, and an example of isomiRs that vary by ends corresponding to miR156#C is shown in panel B. The average abundance of each type of isomiRs is shown in C, and the error bars represent the 95% confidence interval. The star sign indicates the signnificant diffrence (LSD, p <0.05).

As shown in Fig 3 (panel A) and Table 2, both cs-isomiRNA and ls-isomiRNA sequences ranged in length from 18 to 26 nt in length, and sequences 19, 20, 22, and 23 nt in length were a higher percentage of the isomiRNA population than among the miRNAs (Fig 2, panel A compared to Fig 3, panel A) though the percentage of these length classes was lower abundance than the 21 nt and 24 nt isomiRNAs. It should also be noted that like the miRNAs themselves, a higher percentage of ls-isomiRNAs were 24 nt in length compared to the cs-isomiRNAs (26% versus 9.3% respectively).

Compared to their corresponding canonical miRNAs, the isomiRNA sequences varied at either or both ends and can be classified into different categories including: 1) 5’-end variants having different 5’-ends compared to their corresponding canonical miRNAs but having the same 3’-end; 2) 3’-end variants having different 3’-ends compared to their corresponding canonical miRNAs but having the same 5’-end; and 3) 5’- and 3’- variants having different 5’- and 3’-ends compared to their corresponding canonical miRNAs (Fig 3, panel B). IsomiRNAs with variant 3’-ends were the most abundant type (Fig 3, panel C), and were expressed at a significantly higher level (p<0.05), compared to the other two types of isomiRNAs. This is consistent with previous studies that showed most isomiRNAs have the same 5’-end as their corresponding canonical miRNAs.

It has been proposed that DICER or DCL proteins produce isomiRNAs due to the variability of cleavage position during processing of miRNA precursors [64,65]. However, it appears that at least cs-miRNAs and cs-isomiRNAs in *G*. *arboreum* may be produced from multiple genomic loci (S4 Table), and that this phenomenon contributes to the large number of putative cs-isomiRNAs that were observed in this species. It is also worth noting that only 11/156 cs-isomiRNAs (7% of cs-isomiRNAs representing only 1.8% total reads) with expression levels greater than 10 are longer than 22 nt in length (Table 2), and thus, many of the isomiRNAs may be involved in posttranscriptional gene silencing rather than in other types of regulation. The same is not true for the ls-isomiRNAs where over 30% of those sequences are longer than 22 nt in length. However, when only those ls-isomiRNAs with expression of equal to or greater than 10 are considered, only 8.4% of those reads are longer than 22 nt. This allows us to hypothesize that the abundant ls-isomiRNAs are likely functionally important while the low abundance reads appear unlikely to be significant at this time. Further clarification of the *G*. *arboreum* genome is required to conclusively resolve this point and establish a functional role for the abundant number of *G. arboreum* reads with low expression levels.

### Expression analysis of miRNAs and isomiRNAs in different tissues

The expression levels of miRNAs and isomiRNAs varied greatly in different tissues, from zero to over 55,000 RPM (S3 Table). On average, the expression level for cs-miRNAs was 550 RPM/sequence in each tissue library, which was almost 60 times the average expression level of ls-miRNAs (9.8 RPM/sequence). The 1480 cs-isomiRNAs and 553 ls-isomiRNAs observed here had an average expression level of only 53 and 1.4 RPM per sequence (Table 2). However, only 156 cs-isomiRNAs and 12 ls-miRNAs with RPM greater than or equal to 10 accounted for 98% and 97% of all reads respectively. These highly expressed sequences had expression values of 494 and 26 RPM per sequence respectively. The cs-isomiRNAs that were 22 nt in length with expression values greater than or equal to 10 had the highest expression level of any isomiRNAs class (1890 RPM per sequence). This may reflect the fact that miRNAs 22 nt in length are often formed from the same precursors as the predominant 21 nt length class, and likely have related biological functions [12] although they will not be annotated as miRNAs if there is a corresponding sequence 21 nt in length that could be cut from the same sequence.

When the ratio of the expression value of miRNAs versus isomiRNAs was examined, the highest ratio (40.3) was detected in the leaf while the ratio in boll (20.3) and flower (20.1) was lower. However, the expression levels of of the highly expressed isomiRNAs were between 1/3 and 2/3 of the miRNAs. This indicates that the expression of isomiRNAs can is tissue dependent and further supports a functional role for the highly expressed isomiRNAs.

In total, cs-miRNAs versus ls-miRNAs were detected at the highest expression level in the leaf (a total of 100,192 versus 1598 RPM respectively), then at a lower level in the flower (88,902 versus 978 RPM respectively), with bolls demonstrating the lowest expression level (66,602 versus 593 RPM respectively). These data are consistent with the hypothesis that cs-miRNAs may play a critical role in *G*. *arboreum* tissue development as has been shown for other plants [66–68].

**Fig 4:**
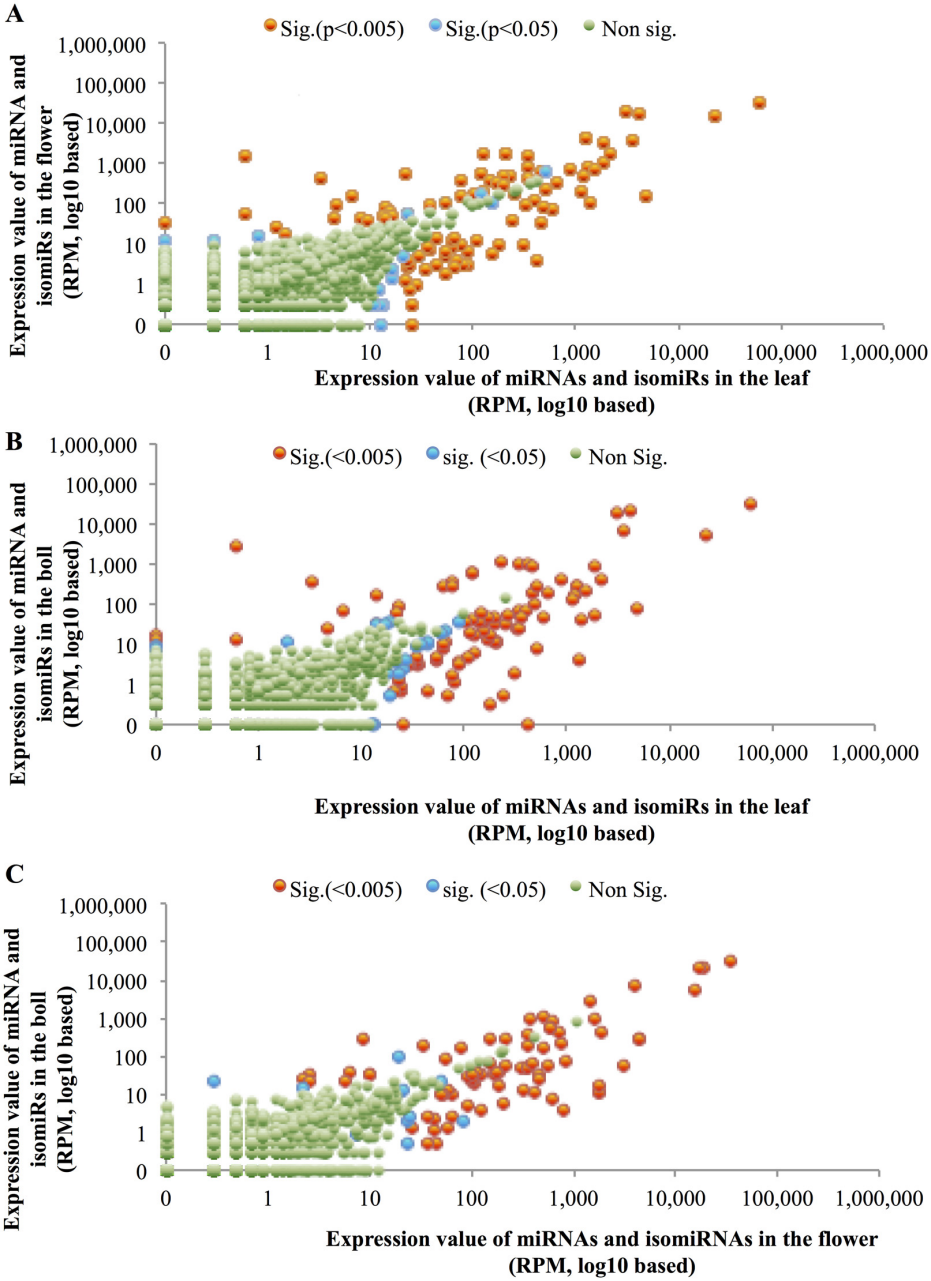
Differentially expressed miRNAs and isomiRNAs between different pairs of tissues. The differentially expressed miRNAs and their isomiRNAs are shown in A (leaf vs. flower), B (leaf vs. boll), and C (flower vs. boll). Red and blue dots indicated sequences that are significantly changed with FDR p-value of < 0.005 and 0.05, respectively, while the light green dots represent mostly low abundance sequences that are not significantly differentially expressed or higher abundance sequences that are not differentially expressed in the tissue comparisons being made.

**Fig 5:**
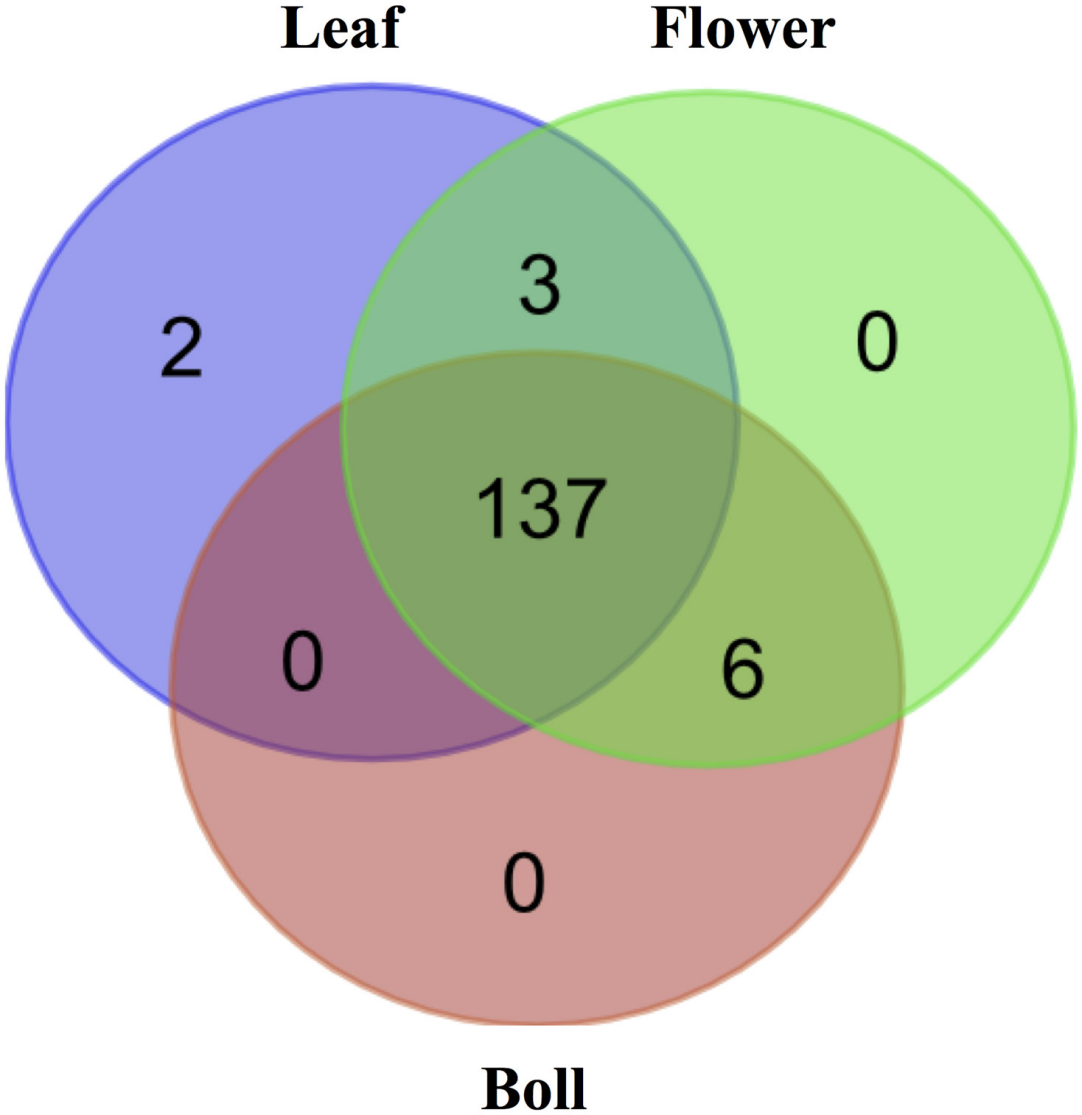
Comparison of differentially expressed miRNAs and isomiRNAs among the three tissues examined. Venn diagram shows the number of differentially expressed miRNAs and isomiRNAs among the three tissue types, leaf, flower and boll as indicated.

To determine differentially expressed miRNAs and isomiRNAs, FDR-corrected (false discovery rate) p-value < 0.05, using Kal’s test (see Methods) was calculated for each pair-wise tissue comparison for each miRNA and isomiRNAs in the dataset (Fig 4 and S4 Table). A total of 148 sequences (7% of the total) were differentially expressed between at least one pair of tissues, including 75 miRNA sequences and 73 isomiRNA sequences. Among those differentially expressed sequences, 89% (131/148) were cs-miRNAs or their isomiRNAs, and only 11% (17/148) were ls-miRNAs or their isomiRNAs. As shown in Fig 5, nearly all (137/148) of the significantly differentially expressed sequences were detected in all three tissue types examined while only 2 sequences were expressed in a single tissue, the leaf. Nine sequences showed joint tissue expression patterns: 6 in flower and boll and 3 in leaf and flower.

Hierarchical cluster analysis of the expression patterns for differentially regulated miRNAs and isomiRNAs showing statistical significance is summarized in Fig 5 and S4 Table. The 148 differentially expressed sequences were clustered into five different groups. Group I consists of 18 sequences (10 miRNAs and 8 isomiRNAs) that are expressed at relatively higher levels in the boll compared to leaf and flower. Group II, contains 13 sequences (7 miRNAs and 6 isomiRNAs) that are more highly expressed in both flower and boll than in the leaf. Group III, is comprised of 34 sequences (17 miRNAs and 17 isomiRNAs) that are expressed at higher levels in the flower than in leaf or boll. Group VI, includes 26 sequences (13 miRNAs and 13 isomiRNAs) that are expressed at higher levels in both leaf and flower than in the boll. Group V, consists of 57 sequences (28 miRNAs and 29 isomiRNAs) that are expressed at higher levels in the leaf than in the other tissues.

Only 4 of the 148 differentially expressed sequences had expression values greater than 10,000 RPM in any one library, while 22 and 65 sequences had expression values greater than 1000 and 100 respectively in any one library. From these highly expressed miRNAs, miR3954#a-b (homologs are found in *Citrus sinensis* L. [69]) was the most abundantly expressed miRNA in all three *G*. *arboreum* tissue types. Hierarchical analysis placed it in Group V indicating a top expression in leaves, decreasing in bolls and flowers. This very high, yet differential tissue expression level suggests an important role for miR3954#a-b over development in *G*. *arboreum* as well as potentially in *C*. *sinensis* where it is also know to occur [69]. Also four isomiRNAs of miR3954#a-b were found, in this analysis (S5 Table). Two of these isomiRNAs, miR3954a18-b15 and miR3954a15-b12, were clustered in Group V with maximum expression values greater than 1500 RPM and less than 60 RPM respectively, while two other cognate isomiRNAs, miR3954a17-b14 and miR3954a30-b27, were found in Group IV showing expression levels over 250 RPM and under 50 RPM, respectively. These 4 isomiRNAs differ in expression largely based on lower (Group V) versus higher (Group IV) expression in flower tissue. There are a total of 10 isomiRNAs with expression values greater than 10 RPM total derived from the 2 miR3954 precursors (S4 Table), and 8 of the 10 isomiRNAs could be cleaved from either the miR3954#a precursor or the miR3954#b precursor. However, two of the isomiRNAs were predicted to only arise from the miR3954#a precursor. These data are consistent with a hypothesis that the miRNA3954#a-b isomiRNAs have different genomic origins, rather than resulting from inconsistent DCL cleavage of the same source.

Among the other highly expressed miRNA sequences were miR167#f-g and its putative cognate isomiRNAs, miR167f10-g10, miR167a4_b4_c4_d4_e2_f9_g9, miR167f14-g14, and miR167a7_f12_g12 are all found in Group II (S5 Table). This miRNA and its isomiRNAs show higher differential expression levels in both flower and boll over leaf, but there are two additional miR167#f-g isomiRNAs that can be observed in Group I, including miR167a6_b7_c7_d5_e4_f11_g11, miR167f8_g8, that demonstrate higher flower expression than in leaf or boll. Because there are six isomiRNAs species with expression levels greater than 10 RPM that are 3’ end variants of miR167#f-g, but there are additional possible genomic sources for at least some of these in the miR167 family of identified putative precursors, it is not possible in this case to demonstrate that differential dicer cleavage cannot account for the isomiRNAs expression patterns we observe.

In Group IV, miR166#a-g was also highly expressed in both leaf and flower but down-regulated in boll. There are a total of 17 putative isomiR167#a-g that demonstrate expression levels greater than 10 RPM (S4 Table). Nine of these were expressed at levels over 100 RPM and were also found in Group IV. However, 5 of the remaining 8 isomiRNAs were not significantly differentially expressed in the datasets while 3 isomiRNAs specific to precursor miR1s66#g were found in Group III (S4 and S5 Tables). These data further support the hypothesis that at least some of the highly expressed isomiRNAs must be derived from differential expression of specific genomic precursors and cannot be explained exclusively by differential dicer cleavage of precursor. Although the functional role of isomiRNAs remains elusive, for at least a subset of isomiRNAs, the analysis done here supports the hypothesis that at least the highly expressed isomiRNAs are involved in the regulation of tissue development in *G. arboreum* as has been shown for development and stress responses in both plants and animals [70,71].

Consistent with previous studies in *Arabidopsis*, rice and upland cotton [36,72–74], *G*. *arboreum* cs-miRNAs were expressed in a similar tissue specific manner. For example, miR156 and miR535 are expressed at higher levels in leaf compared to flower and boll, in comparison to other families, such as miR166, miR167, miR390, and miR171 that were preferentially expressed in flower or boll. Also in *Gossypium hirsutum* miR2949 and miR2950 are similarly expressed compared to *G*. *arboreum* [36].

### Targets of *G*. *arboreum* miRNAs

A total of 8,184 putative target sequences were predicted in the *G*. *arboreum* genome assemblies and EST sets for 259 of the 263 unique mature miRNAs and for 1,756 of the 1,840 isomiRNAs (Table 3, S6 Table) using the psRNA Target utility [41]. However, no sequences were found in the database that appeared to be targets for 4 miRNAs and 84 isomiRNAs (Table 3). Targets were predicted for cs-miRNAs and cs-isomiRNAs (1659 and 3229 targets, respectively), and for ls-miRNAs and ls-isomiRNAs (1364 and 2310 targets, respectively). Overall just under 30% of the targets were shared between miRNAs and their cognate isomiRNAs, and this also applies to both cs-miRNAs/isomiRNAs or ls-miRNAs/isomiRNAs. Only 11% and 22% of cs-miRNA or ls-miRNA targets, respectively, were unique to the miRNAs while approximately 70% of the targets were unique to isomiRNAs. Thus, a majority of lineage-specific isomiRNA targets can be predicted by considering isomiRNAs distinctly from miRNAs. This supports the hypothesis that isomiRNAs frequently have different and more numerous targets than their cognate miRNAs based on these predictions. Consistent with these prediction is the hypothesis that isomiRNAs have different, likely broader, functional roles than their cognate miRNAs as has been found in previous studies [60,75,76].

**Table 3:** Summary of miRNA and isomiRNA target information from S6 Table.

	miRNA	isomiRNA	unique	
	targets	targets	mature	No Targets
Total				
miRNA	2934		263	4
isomiRNA				
All		5250	1840	84
Exp >10 RPM		740	152	
cs-miRNA	1659		155	4
cs-isomiRNA				
All		3229	1286	37
Exp >10 RPM		633	138	
ls-miRNA	1364		108	0
ls-isomiRNA				
All		2310	554	47
Exp >10 RPM		112	14	

The possible biological relevance of miRNA/isomiRNA target predictions was further assessed by a comparison of the targets for only those sRNAs derived from miRNAs or isomiRNAs that demonstrated significant expression-level changes in one or more tissue comparisons above. For the 148 differentially expressed miRNAs/isomiRNAs in one or more pairs of tissues, a total of 1,821 target genes were predicted, of which 1,279 and 563 targets were found for 110 cs-miRNAs/isomiRNAs and 32 ls-miRNAs/isomiRNAs, respectively (Table 4). For these targets 28% (353/1,279) were shared between cs-miRNAs and their isomiRNAs, whereas only 13% (73/563) were shared between ls-isomiRNAs and their cognate ls-miRNAs. Thus, at least half of the isomiRNA targets are unique to the isomiRNAs, while 63% of the cs-miRNA targets and 85% of the ls-miRNA targets were unique to the miRNAs and not shared as targets among the isomiRNA and miRNAs (Table 4).

**Table 4:** Summary analysis for predicted targets of differentially expressed *G*. *arboreum* miRNA.

4A. Total *G*. *arboreum* miRNAs	cs-miRNAs	cs-isomiRNA	ls-miRNAs	ls-isomiRNA
**1. Total number of miRNA/isomiRNA**	**155**	**1286**	**108**	**554**
**2. Number having targets**	151		108	507
**3. Number of targets**	**1659**	**3229**	**1364**	**2310**
**4. Targets Shared miRNAs & isomiRNAs**		1083		802
**5. Percent unshared targets**	34.7%	66.5%	41.2%	65.3%
**6. Targets shared by cs- and ls-miRNAs**			356	
**7. Total unique targets**			6321	
**4B. Upregulated miRNAs**	**cs-miRNAs**	**cs-isomiRNA**	**ls-miRNAs**	**ls-isomiRNA**
**1. Total number of miRNA/isomiRNA**	**53**	**63**	**22**	**10**
**2. Number having targets**	53	57	22	10
**3. Number of targets**	**976**	**656**	**488**	**148**
**4. Targets Shared miRNAs & isomiRNAs**		353		73
**5. Percent unshared targets**	63.8%	46.2%	85.0%	50.7%
**6. Targets shared by cs- and ls-miRNAs**	21			
**7. Total unique targets**	1821			

By comparison, less than 35 and 42% of all predicted cs- and ls-miRNA targets respectively are unique to miRNAs while the more than 65% of predicted cognate cs- and ls-isomiRNA targets are unique (Table 4). However, for the highly expressed genes (Table 4) a much higher percentage of predicted miRNA targets are unique (63–85%) compared to ~50% unique targets for isomiRNAs (Table 4). This strengthens support for the concept that differentially expressed isomiRNAs can play a biologically significant role in regulating different target genes than their cognate miRNAs, and is consistent with previous studies showing roles for isomiRNAs in tissue development [60,75,76].

### Pha-siRNAs in *G*. *arboreum*

Using the bioinformatics pipeline outlined in S3 Fig, 319 phased-siRNA-producing gene transcripts (PGTs) were predicted. From these PGTs, 833 putative pha-siRNAs were identified in the *G*. *arboreum* sRNA datasets, and an additional 2,382 putative non-phased siRNAs (non-pha-siRNAs) were predicted deriving from the 319 PGTs (S7 and S8 Tables).

The biogenesis of pha-siRNAs (ta-siRNAs) has been shown to be a stepwise process, in which PGTs (TAS genes) are first cleaved by miRNAs or other siRNAs, and subsequently double-stranded RNAs are synthesized with the assistance of SGS3 (Suppressor of Gene Silencing 3) and RDR6 (RNA Dependent RNA polymerase 6) at the initiator site (trigger-site) created by miRNA- or siRNA-directed cleavage [26,77,78]. Table 5 shows that approximately 87% of all PGTs (278/319) were predicted to be triggered by 90 putative miRNAs, 420 putative isomiRNAs, and/or 1161 putative pha-siRNA triggers (S7 Table and Table 5) while the remaining 41 PGTs have no presently discernable trigger. The complexity of secondary RNA production is easily demonstrated by considering that of the 278 putative triggered PGTs only 70 are triggered by an average of just over 1 miRNA (52 miRNA triggered PGTs have only 1 miRNA trigger, 16 have 2 distinct miRNA triggers, and but 2 have 3 distinct miRNA triggers), while 149 PGTs have an average of 2.8 isomiRNA triggers, and 262 of the 278 PGTs can be triggered by as many as 1161 pha-siRNAs having an average of 4.4 triggers each (Table 5). While it is true that the putative miRNA triggers are more highly expressed (average 879 RPM, Table 5), many of the isomiRNAs triggers are also expressed at meaningful levels, making it likely that this group has high impact on secondary siRNA production. Although the pha-siRNA triggers are expressed at dramatically lower levels (average 3.2 RPM per trigger, Table 5) than the other trigger classes, it is clear that there are a number of pha-siRNA triggers with meaningful expression levels making many pha-siRNAs capable of acting as triggers for pha-siRNA production as will be discussed below.

**Table 5:** Summary Table of secondary siRNAs from *G*. *aroboreum* from PGTs with and without triggers.

	PGTs	PGTs by trigger type	Trigger Number	Average Triggers/PGT	Average RPM/ Trigger	Triggers with RPM > 1
**Total PGTs**	319					
**PGTs with triggers**	278		1671	6.01		
** miRNA**	1	70	90	1.29	879.2	90
** miRNA & pha-siRNA**	8				(RPM Range—2.1–37835.9)	
** miRNA or isomiRNA**	8					
** miRNA, isomiRNA, & pha- siRNA**	53					
** isomiRNA**	7	149	420	2.82	127.1	171
** isomiRNA & pha-siRNA**	81				(RPM Range—0.4–37772.5)	
** pha-siRNA**	120	262	1161	4.43	3.2	620
**PGTs without triggers**	41				(RPM Range—0.5–205.7)	

While bioinformatics prediction of PGTs are likely incomplete, the predictions made here are consistent with miRNAs as well as both isomiRNAs and/or phased/non-phased siRNAs being triggers for the biogenesis of secondary-siRNAs in *G*. *arboreum*. This is further supported because the 755 total pha-siRNAs and 2251 total unpha-siRNAs have expression values averaging 8.5 and 4.1 RPM respectively (Table 6). While these are meaningful levels of expression it was also observed that 700 pha-siRNAs and 984 unpha-siRNAs with expression levels greater than or equal to 1 RPM had average expression levels of 24.1 and 18.5 RPM per siRNA respectively (Table 6). The corresponding average expression values for the siRNAs deriving from the PGTs without triggers is about 1/3 the values for the siRNAs derived from the triggered PGTs. Thus, based on expression values it appears that a most of the phased and unphased siRNAs that are expressed at biologically meaningful levels have been recovered in this analysis and the majority of these are derived from triggered PGTs.

**Table 6:** Summary of secondary siRNA production from PGTs derived from *G. arboreum* siRNA libraries.

	Total		Expression >1 RPM	
	phased	unphased	phased	unphased
**Triggered**				
** Total siRNAs**	755	2251	700	984
** PGTs**	278	270	278	197
** Aveage/PGT**	2.7	8.3	2.5	5.0
** Average RPM/siRNA**	8.5	4.1	24.1	18.5
** RPM range**	0.5–8921	0.5–2581		
**Without Triggers**				
** Total siRNAs**	78	131	71	81
** PGTs**	40	40	40	34
** Aveage/PGT**	2.0	3.3	1.8	2.4
** Average RPM/siRNA**	2.5	3.5	8.1	6.5
** RPM range**	0.5–78.5	0.5–157.3		

It is also apparent most PGTs appear to be triggered by multiple miRNAs, isomiRNAs, or siRNAs in several different positions, reflecting that the complexity of secondary-siRNA production in *G*. *arboreum* is consistent with or even expands our understanding derived from observations in other model species [3].

**Fig 6:**
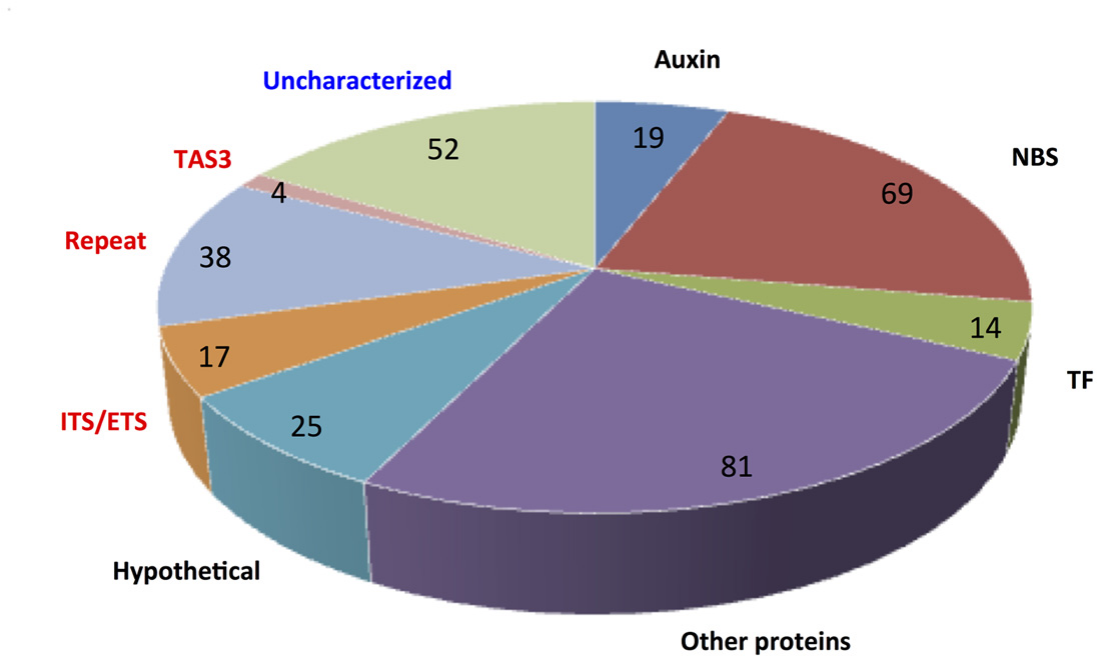
Categories of pha-siRNA-yielding genes. Categories and total number of pha-siRNA-producing genes in each category are shown. Based on BLASTX and BLASTN searches categories can be further specified as containing protein coding sequences (black text), uncharacterized sequences (blue text), and non-protein coding sequences (red text). Specific subcategories include the following: Auxin: represents genes that encode Auxin-related proteins, including Auxin Response Factors (ARF) and Auxin Signaling F-box. NBS: represents genes that encode NBS-LRR disease resistant genes; TF: represents genes that encode transcription factors, including MYB and bHLH. Other proteins: represents genes that encode other proteins, including kinases and ATPases. Hypothetical: represents genes that encode unknown protein products; ITS/ETS: represents genes that encode ribosomal internal/external transcribed spacers (ITS/ETS); Repeat: genes that are derived from repeat sequences; TAS3: Arabidopsis TAS3-homologs; Uncharacterized: represents genes that are not homologue any known genes or proteins.

The 319 PGTs found in this study consisted of 208 putative protein-coding genes, 59 non-protein-coding genes, and 52 presently uncharacterized genes (Fig 6). Among the genes characterized as protein-coding-gene-derived putative PGTs by BLASTX and/or BLASTN searches (Fig 6), 69 proteins encode apparent NBS-LRR disease resistance proteins orthologs; 19 PGTs corresponded to Auxin-related protein orthologs, including Auxin Response Factors and Auxin Signaling F-box proteins; and 14 PGTs encoded apparent transcription factors. A large group of PGTs (81) encode other defined proteins, including DCL proteins and genes likely involved in metabolism, as well as 25 hypothetical proteins. Additionally, a number of PGTs were derived from non-protein-coding genes, including 4 Arabidopsis *TAS*3 homologs, 17 internal/external transcribed spacers of ribosomal RNAs (ITS/ETS), and 38 repeat DNA sequences.

Conserved PGTs, having previously defined functions including, *TAS*3-homlogs and NBS-LRR disease resistance proteins, were reported to be triggered by conserved miRNAs such as miR390 and miR482, respectively [32,79]. The same conserved *G*. *arboreum* miRNA orthologs also trigger secondary siRNA production from these *G*. *arboreum TAS*3 and NBS-LRR orthologs. S7 Table also shows that there are at least 20 additional *G*. *arboreum* PGTs that are known targets of the same cs-miRNAs in other species although only the TAS3-homologs and the NBS-LRR proteins have previously been shown to be a source of secondary-siRNAs [28,32]. Additionally, there are 53 PGTs triggered by one or more cs-miRNAs that have not previously been shown to be PGTs or targets of their respective miRNA triggers. These PGTs consist of multiple members of 13 different protein-coding gene families including 16 NBS-LRR proteins triggered by cs-miRNAs not previously known to trigger NBS-LRR proteins in other systems.

There were also at least 12 PGTs discovered as targets of cs-miRNAs that have not previously been reported as PGTs along with multiple hypothetical uncharacterized protein-coding sequences and at least 6 repeat-element associated sequences producing secondary siRNAs. The protein coding sequences (S7 Table) include: alcohol dehydrogenases, ARF1, arginine N-methyltransferase, asparagine synthase, chaperonin 60, S-adenosylmethionine decarboxylase, and a zinc finger protein. This diversity of novel putative secondary-siRNA-producing gene transcripts triggered by plant cs-miRNAs, as well as the predicted complexity of the *G*. *arboreum* known secondary-siRNA-producing pathways compared to model species suggests that the complexity of secondary siRNA pathways may be far more intricate than previously envisioned, and that such pathways will likely demonstrate a significant evolutionary diversity even between closely related species.

### *Cis*- and *trans*-cleavage of secondary siRNAs

More recently, it has been shown that PGTs can be processed by so-called “*cis*-cleavage”, seemingly by-passing miRNA-directed initiation of pha-siRNA production [3]. These *cis*-cleavage products are derived from PGTs, but were found as siRNAs showing phased patterns different from the phasing patterns produced by miRNA-triggered siRNAs [3] coming from the same PGT. Thus, *cis*-cleaved siRNAs are among those that were characterized as non-phased siRNAs in this study, but they derive from PGTs the same PGT as pha-siRNAs but with different phasing. In this study, 1,699 non-phased secondary siRNAs were observed, of which 835 sequences from 69 PGTs appeared to be possible products of *cis*-cleavage by 209 phased/non-phased siRNAs derived from the same PGTs (S9 Table). In addition, 511 sequences derived from 105 PGTs were predicted for cleavage by 108 phased/non-phased siRNAs derived from other PGTs or by 39 miRNAs/isomiRNAs not known to produce pha-siRNAs (S10 Table). Because of the apparent similarity to *cis*-cleaved siRNAs, but the fact that these putatively phased siRNAs function on other PGTs, this subset of putative phased siRNAs will be referred to as *trans*-cleaved siRNAs. Interestingly, 43 PGTs could be cleaved by both *cis*- and *trans*-cleavage (S9 and S10 Tables).

The importance of potential *cis*- or *trans*- cleavage of apparently non-phased siRNAs also derives from a consideration of the expression values of secondary siRNAs produced from such putative cleavage products. The *cis*-cleaved pha-siRNAs have on average 29 total RPM, and there are 421 pha-siRNAs with RPM greater than 100 (Table 7, panel A). Also note that the average expression changes between tissues suggesting that there may be tissue specific expression changes in these species of siRNA. The *trans*-cleaved pha-siRNAs have on average only 4.2 total RPM, and only 2 of these have total RPM greater than 100 although 50 have total RPM greater than 10 (Table 7, panel B), and although there are fewer of these overall an even smaller percentage of these are expressed at biologically meaningful levels. However, there are at least some of these that are expressed at such levels. Thus, a number of cis-cleaved pha-siRNA and at least some of the trans-cleaved pha-siRNAs with expression levels high enough to produce meaningful effects on posttranscriptional gene regulation.

**Table 7:** Expression summary table for cis-cleaved and trans-cleaved secondary-siRNA. Derived from S9 and S10 Tables.

	Leaf	Flower	Boll	Total
**A. *cis*-cleaved siRNA expression**				
siRNAs Number—total	6991	6991	6991	6991
siRNAs Number—Exprression > = 1 RPM	2914	3602	2150	4970
siRNAs Number—Exprression—> = 10 RPM	913	1167	574	1771
siRNAs Number—Exprression > = 100 RPM	169	152	75	421
Average RPM—total	9.8	13.8	5.8	29.4
Average RPM—Expression > = 1 RPM	23.2	26.4	18.0	41.0
Average RPM—Expression > = 10 RPM	67.5	74.0	57.4	108.9
Average RPM—Expression > = 100 RPM	251.9	348.0	218.5	357.7
Maximum	693.7	1489.7	397.5	2580.9
Minimum	0.1	0.1	0.1	0.5
**B. *trans*-cleaved siRNA Expression**				
siRNAs Number—total	1012	1012	1012	1012
siRNAs Number—Exprression > = 1 RPM	137	130	146	417
siRNAs Number—Exprression—> = 10 RPM	16	10	18	50
siRNAs Number—Exprression > = 100 RPM	2	2	1	2
Average RPM—total	1.5	1.7	1.1	4.2
Average RPM—Expression > = 1 RPM	9.3	11.2	5.8	9.3
Average RPM—Expression > = 10 RPM	59.2	115.2	31.6	61.0
Average RPM—Expression > = 100 RPM	323.4	511.8	203.0	941.1
Maximum	352.1	730.3	203.0	1285.4
Minimum	0.1	0.1	0.1	0.5

Thus, a large number of non-phased siRNAs (1088/1699) appeared to be cleaved by so-called *cis*-cleavage or by trans-cleavage, supporting the hypothesis that the biogenesis of non-phased siRNAs through non-canonical cleavage appears to be widespread and is likely meaningful in at least *G*. *arboreum* as has been found for model species [3].

#### Expression of pha-siRNAs, and *cis*- and *trans*-cleaved secondary siRNAs

**Fig 7:**
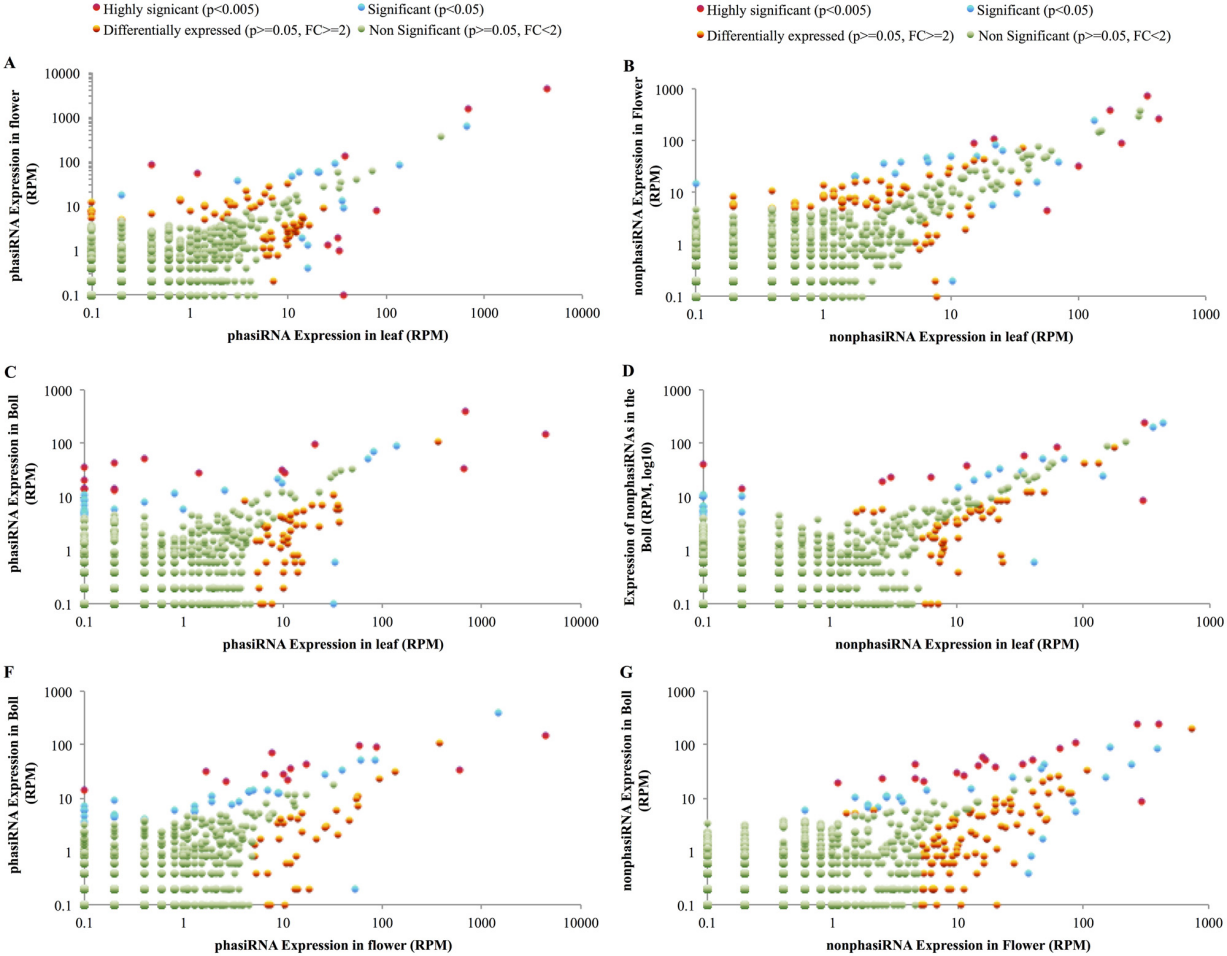
Expression changes of phased and non-phased siRNAs among the three tissues examined. The expression changes of phased and non-phased siRNAs between different tissues are shown. The expression changes for phasiRNAs are shown in Panel A, C, and E, and the expression changes for non-phased siRNAs are shown in Panel B, D, and E. The siRNA sequences with p< 0.005 and p<0.05 are shown in red and blue, respectively. Those sequences with FDR p ≥0.05, which are expressed at higher levels (≥5 RPM) and are regulated at least 2 fold changes, are shown in orange, while the siRNA sequences with FDR p ≥0.05, which are not expressed at higher levels (< 5RPM) or are not regulated less 2 fold changes, are shown in green.

To determine differential expression of phased and non-phased siRNAs, Kal’s test was employed to analyze the expression changes of pha-siRNAs, and *cis*- or *trans*- cleaved non-pha-siRNAs. As a result, 60 phased and 58 non-phased siRNAs (approximately 5% of the total) were differentially expressed demonstrating FDR-corrected p-values < 0.05 between at least one pair of tissues (Fig 7, S10 Table). These pha- and nonpha-siRNAs were produced from 49 and 83 PGTs respectively. Additionally, 57 phased and 99 non-phased siRNAs that were expressed at levels ≥ 5 RPM in at least one tissue and their expression was up- or down-regulated by at least 2-fold in at least one pair of tissues were placed in a category of likely differentially expressed si-RNAs although this differential expression was not statistically significant at the p > 0.05 (Fig 7, S10 Table).

These likely differentially expressed pha- and non-pha-siRNAs were produced from 119 and 60 PGTs respectively (Table 8, panel A). Of the potential to triggers of PGT-cleavage described above, 13 miRNAs, 13 isomiRNAs, 13 phased-siRNAs, and 10 non-phased siRNA triggers were differentially expressed (data not shown), and these triggers lead to the production of secondary siRNAs from 54 PGTs. Note that all of the PGTs triggered by differentially expressed triggers produce secondary siRNAs that are differentially expressed although the tissue-specific pattern of secondary siRNA differential expression frequently varies from the tissue-specific expression pattern of the trigger. This describes yet another level of complexity required to explain the diversity of such results, that likely derives from differences in the regulation of transcript synthesis as well as differential effects of precursor cleavage and processing of siRNAs from the double stranded complexes.

**Table 8:** Summary of significantly differentially expressed siRNAs and likely differentially expressed siRNAs and their triggers.

A. P-value & fold change from *G. arboreum* leaf, flower, and boll comparisons.				
	FDR p < 0.05		Fold change ≥2	
	PGT for phasiRNAs	PGT for non-phasiRNAs	PGT for phasiRNAs	PGT for non-phasiRNAs
**PGTs**	49	83	22	54
**Shared (phased & non-phased)**	13		16	
**Total**	119		60	
**Shared (p-value & fold change)**	34			
**Total**	145			
**B. Based on fold change from *G. arboreum* leaf, flower, and boll comparisons shown by source of trigger for siRNA production.**				
	**Differentially Expressed Triggers**			
	**miRNA**	**isomiRNA**	**pha-siRNA**	**nonpha-siRNA**
**PGTs**	24	39	21	15
**Shared (phased & non-phased)**	9		9	
**Total**	54		27	
**Shared (p-value & fold change)**	27			
**Total**	54			

**Fig 8:**
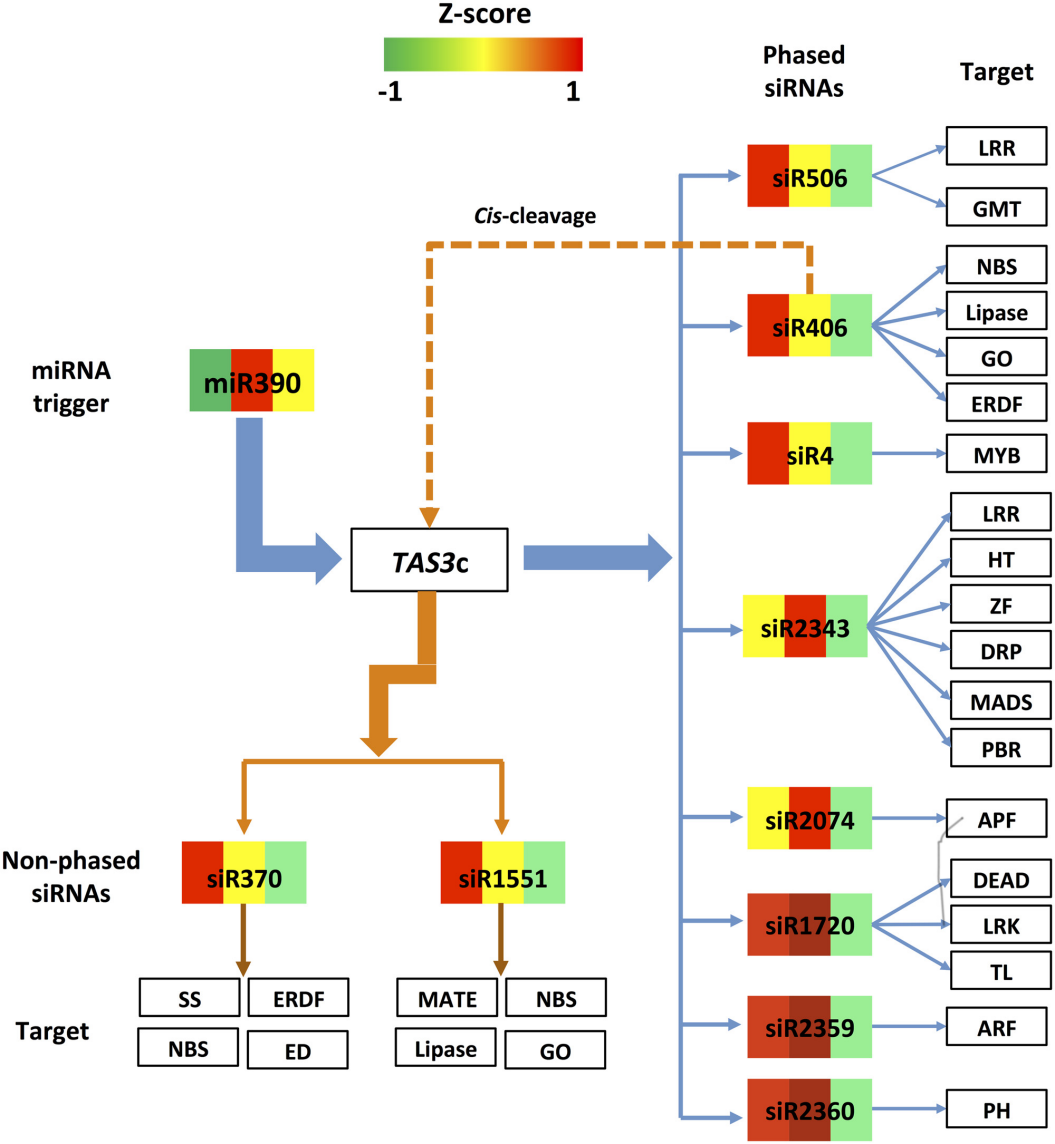
miR390-directed pha-siRNA regulatory pathway. The miR390 directed pha-siRNA regulatory pathway is shown as indicated. The blue arrows and lines show the regulatory pathway of pha-siRNA s produced from TAS3c, while the dark orange arrows and lines show the regulatory pathway of cis-cleavage by pha-siRNA, siR406. The colors in the boxes from left to right represent the Z-score of expression values of sRNAs in the leaf, flower and boll, respectively. LRR: Leucine-rich repeat family protein; GMT: DP-mannose transporter; NBS: NBS-LRR disease resistance protein; Lipase: Lipase; GO: Glycolate oxidase; ERDF: Early-responsive to dehydration family protein; MYB: MYB transcription factor; HT: hexose transporter; ZF: Zinc finger family protein; DRP: DNA-directed RNA polymerase family protein; MADS: MADS-box transcription factor; PRP: Phenylcoumaran benzylic ether reductase-like protein; APF: Amino acid permease family protein; DEAD: DEAD-box ATP-dependent RNA helicase; LRK: LysM type receptor kinase; TL: Temperature-induced lipocalin; ARF: Auxin response factor; PH: Pleckstrin homology domain-containing family protein; SS: Spermidine synthase; ED: Elongation defective 1 family protein; MATE: MATE efflux family protein.

**Fig 9:**
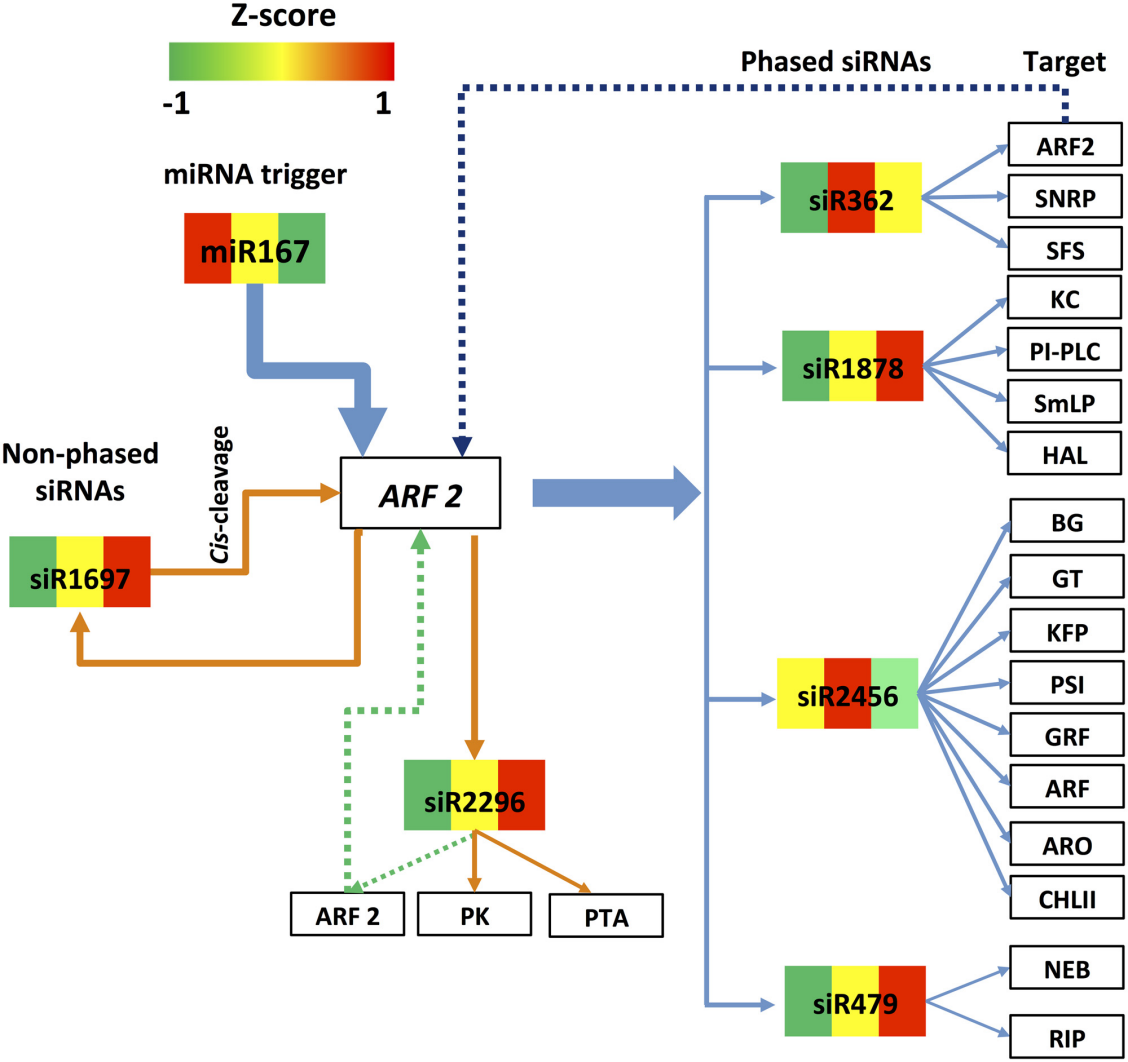
miR167-directed pha-siRNA regulatory pathway. The miR167 directed pha-siRNA regulatory pathway is shown as indicated. The blue arrows and lines show the regulatory pathway of pha-siRNA s produced from ARF2, while the dark orange arrows and lines show the regulatory pathway of cis-cleavage by nonpha-siRNA, siR1697. The broken green and dark blue arrows show two potential auto-regulated pathway of Auxin by phased and non-phased siRNAs. The colors in the boxes from left to right represent the Z-score of expression values of sRNAs in the leaf, flower and boll, respectively. ARF (2): Auxin response factor (2); SNRP: small nucleolar RNA-associated protein; SFS: Splicing factor subunit; KC: kafirin cluster; PI-PLC: PI-PLC X domain-containing protein; SmLP: snRNA-associated Sm-like protein; HAL: PAP-specific phosphatase HAL2-like family protein; BG: Beta-galactosidase; GT: Glycosyltransferase; KFP: Kinase family protein; PSI: Photosystem I chain III family protein; GRF: GRF1-INTERACTING FACTOR 2 family protein; ARO: ARO1-like protein; CHLII: hlorophyll A/B binding protein; NEB: Neurobeachin; RIP: 60S ribosomal protein L5.

**Fig 10:**
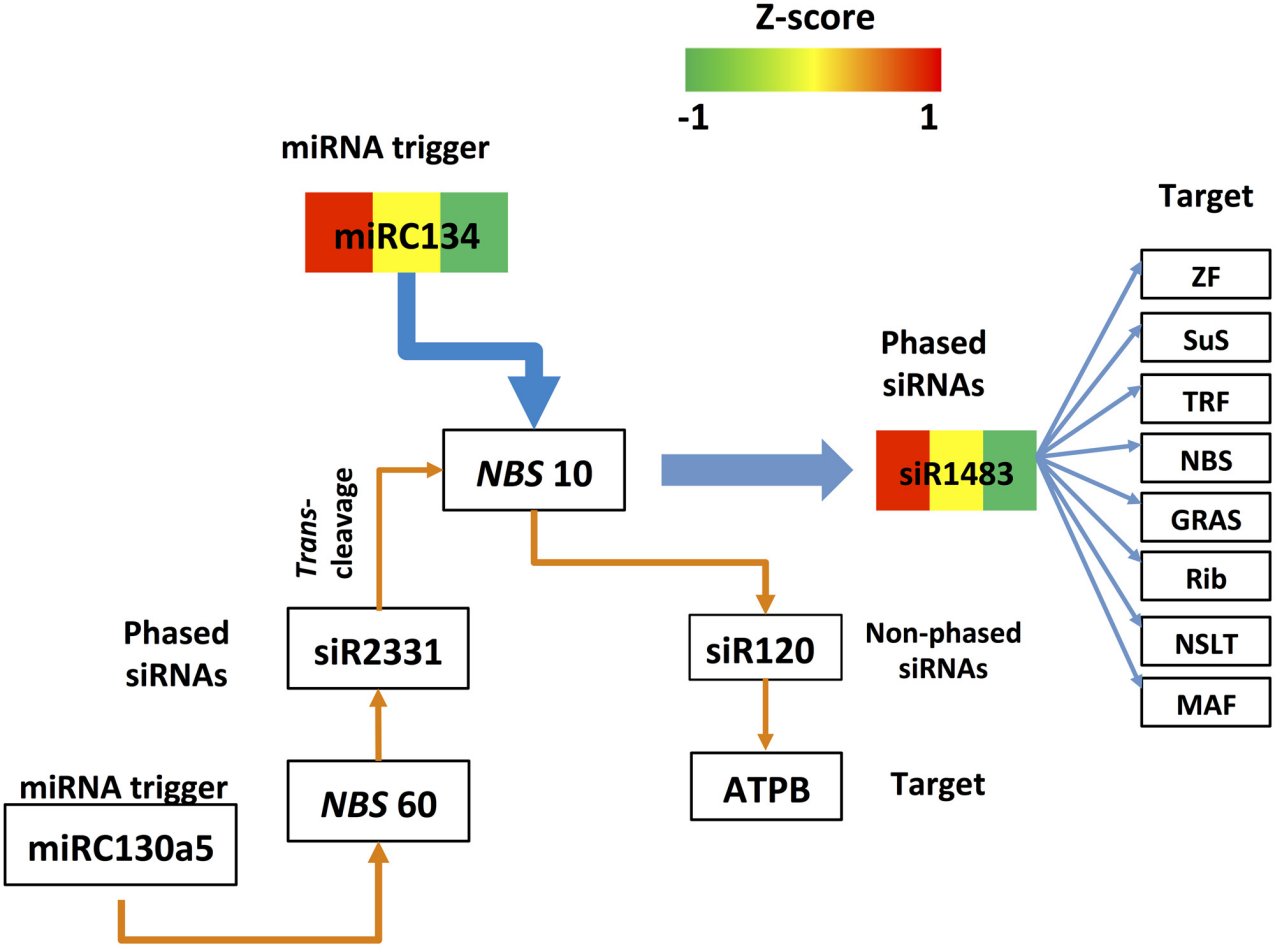
A pha-siRNA regulatory pathway that is co-regulated by miRC134 and pha-siR2331. The pathway of pha-siRNAs derived from NBS 10 that is co-regulated by miRC134 and phased siRNAs, siR2331, is shown as indicated. The blue arrows and lines show the regulatory pathway of pha-siRNAs produced from NBS 10, while the dark orange arrows and lines show the regulatory pathway of trans-cleavage by pha-siRNA, siR2331, which is derived from NBS 60. The colors in the boxes from left to right represent the Z-score of expression values of sRNAs in the leaf, flower and boll, respectively, while miRC130a5, siR2331, and siR120, are not differentially expressed in the tissues exampled. NBS 10 or 60: NBS-LRR disease resistance protein 10 or 60; ZF: Zinc finger protein; SuS: sucrose synthase; TRF: Transducin-related family protein; GRAS: GRAS family protein; Rib: Ribophorin 1 family protein; NSLT: Non-specific lipid-transfer protein; MAF: Mitochondrial carrier family protein; ATPB: ATP binding protein.

It is clear that sRNA regulatory networks have the ability to play important regulatory roles in tissue differentiation, but the details for such regulation require further study as well as experimental validation at this juncture. However, some examples can be described and are shown in Figs 8, 9 and 10.

#### Target prediction for pha-siRNAs, and nonpha-siRNA

**Fig 11:**
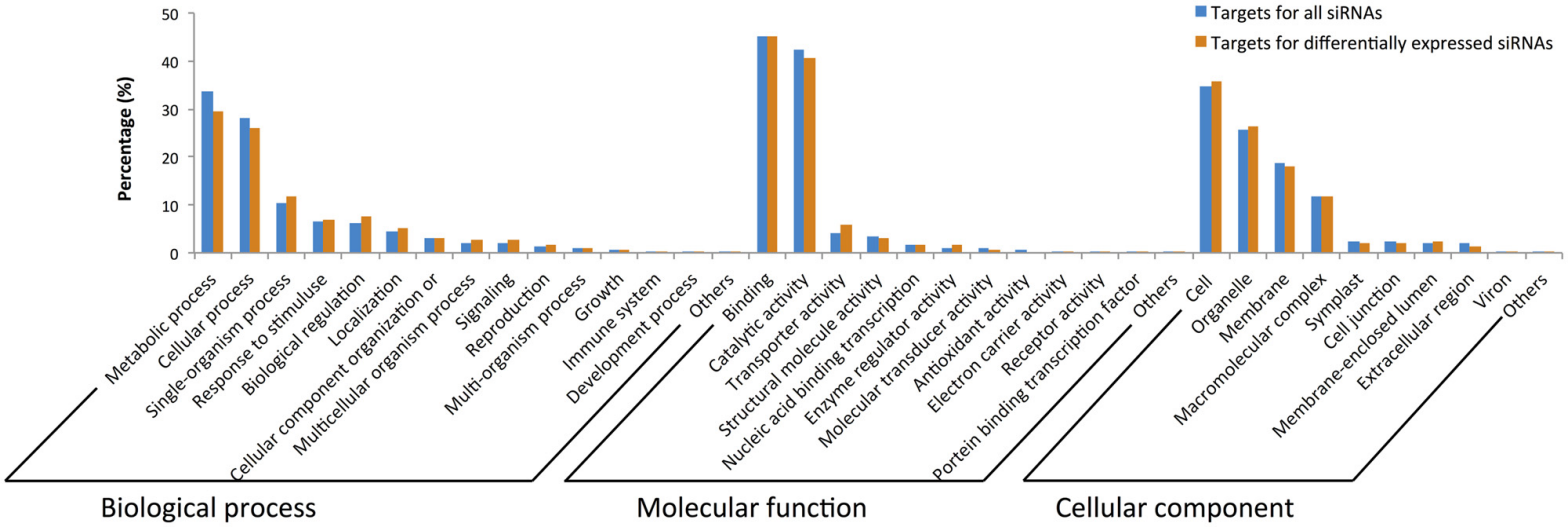
GO analysis of targets of phased and non-phased siRNAs. The GO analysis of targets of phased and non-phased siRNAs is shown, and GO terms are grouped by Biological process, Molecular function, and cellular component. Targets of all secondary siRNAs are shown in blue, while targets of differentially expressed siRNAs are shown in dark orange.

Target analysis of phased and non-phased siRNAs revealed that 13,128 genes for 2,555/2,655 pha-siRNAs and nonpha-siRNAs, respectively, were found (S7 Table), but targets were not predicted for 100 non-phased siRNAs. Predicted putative targets included transcription factors, such as bHLH (Basic Helix-loop-helix), MYB-type TFs, auxin response factors (ARF), and ethylene response factors (ERF) or enzyme involved in signaling (phosphatidylinositol 3- and 4-kinase) or cellular processes (calcium ATPase). Fig 11 shows the GO analysis of the 13,128 predicted targets of the secondary siRNAs. Note that the information reported in the figure for secondary siRNAs is not strikingly different from that of miRNAs. Interestingly, the pha-siRNAs derived from an Auxin Signaling F-box (ASF) was predicted to target Auxin Response Factor, which suggested an auxin-regulated siRNA auto-regulatory network [32].

Targets were also examined for the differentially expressed and likely differentially expressed pha-siRNAs and non-phased siRNAs. This yielded 915 and 803 targets for 117 phased and 157 non-phased siRNAs, respectively, and 138 targets were shared between phased and non-phased siRNAs. Go analysis of these targets essentially paralleled the types of targets predicted for total secondary siRNAs (Fig 11).

## Conclusion

In this study, an extensive investigation of miRNAs, isomiRNAs, pha-siRNAs, and cis- and trans-cleaved pha-siRNAs was conducted by analyzing deep sequencing small RNA libraries generated from *G*. *arboreum* leaf, flower and boll. This revealed that a large number of conserved and lineage-specific miRNAs and secondary-siRNAs were encoded in the genome of *G*. *arboreum*. Both isomiRNAs and non-phased siRNAs were observed along with their cognate miRNAs and pha-siRNAs respectively. This is consistent with previous studies showing that non-phased siRNAs can be produced by so-called *cis*-cleavage and also by what we describe here as *trans*-cleavage. In our studies it appears that more non-phased siRNAs were derived from *trans*-cleavage than from *cis*-cleavage. Expression analysis showed that a subset of those sRNAs were significantly regulated in one or more pairs of tissues examined, which is consistent with their regulatory roles in tissue development. Those sRNAs were predicted to regulate approximately thirteen thousand *G*. *arboreum* genes that are involved in a broad spectrum of metabolic, cellular, and enzymatic activities that are expected to be associated with tissue development implying that sRNA regulatory networks play a critical role in regulating development.

Homologs of relatively conserved sRNA regulatory networks elucidated in model organisms also exist in *G*. *arboreum*, although the details of such networks may differ in major ways. Additionally, specific sRNA regulatory pathways and parts of conserved pathways that are lineage-specific to *G*. *arboreum* and that involve both conserved and lineage-specific miRNAs/isomiRNAs can readily be identified from such bioinformatics analysis.

Results from this study significantly extended our knowledge of the scope of sRNA families in the regulation of tissue development, and clearly demonstrate the need to further elucidate and characterize specific pathways while at the szme time recognizing that more complexity exists that implified models can account for. A further, more thorough analysis of the *G*. *arboreum* genome, and a more extensive EST dataset with which to work will greatly expedite this work.

## Supporting Information

S1 Fig Bioinformatics pipeline for overall prediction of miRNA and pha-siRNAs in *G*. *arboreum*. Pipeline description (shown on left) and the resulting number of sequences predicted in each stage of the pipeline (shown on right). Number of total sRNA reads (black); unique sRNA sequences (blue).
(TIFF)

S2 Fig Bioinformatics pipeline for prediction of miRNA candidates in *G. arboreum*. Pipleine description (shown on left) and the resulting number of sequences predicted in each stage of the pipeline (shown on right). Number of unique sRNA sequences (black); miRNA gene precursor sequences (blue). Novel lineage specific miRNA candidates were found in *G*. *aroborem* but not in other plants.
(TIFF)

S3 Fig Bioinformatics pipeline for prediction of phasiRNAs in *G*. *arboreum*. Pipeline description (shown on left) and the resulting number of sequences predicted in each stage of the pipeline (shown on right). Number of unique sRNA sequences (black); Pha-siRNA gene transcripts (blue).
(TIFF)

S1 Table Initial processing read statistics for leaf, flower, boll and total libraries. (XLSX)

S2 Table Summary of information for *G*. *arboreum* conserved and lineage-specific miRNAs and their precursor sequences. (XLSX)

S3 Table List of miRNAs and their corresponding isomiRs found in *G. arboreum* leaf, flower and boll tissues. (XLSX)

S4 Table Differentially expressed *G. arboreum* miRNAs and isomiRNAs based on significant Kal's test and fold change in expression in the 3 tissue comparisons. (XLSX)

S5 Table Comparison of highly expressed miRNAs and isomiRNAs found in the *G. arboreum* genome. (XLSX)

S6 Table List of predicted targets for *G*. *arboreum* miRNAs and isomiRNAs with descriptions. (XLSX)

S7 Table List of the 278 G. raimondii PGTs that have triggers showing expression levels of all triggers and siRNAs, and summary information for targets of the Phased and unphased secondary siRNAs produced. (XLSX)

S8 Table List of the 41 G. raimondii PGTs without triggers showing expression levels of the phased and unphased secondary siRNAs produced and summary information for targets of the untriggered phased and unphased secondary siRNAs. (XLSX)

S9 Table cis-cleaved PGT analysis. (XLSX)

S10 Table trans-cleaved PGT producing siRNAs. (XLSX)

S11 Table Differential expression analysis of phased- and non-phased siRNAs showing FDR-corrected p-values for pairwise comparisons of leaf, flower and boll tissues. Block shown in red are significantly differentially expressed (FDR-adjusted p-value <0.05 in at least one comparison). Blocks shown in blue include likely differentially expressed sequences (Fold change>2, FDR p-value > 0.05 in at least one comparison).
(XLSX)
