# Supplementary figures and images for: The Complexity of Posttranscriptional Small RNA Regulatory Networks Revealed by In Silico Analysis of Gossypium arboreum L. Leaf, Flower and Boll Small Regulatory RNAs
Source: PLoS One. 2015 Jun 12;10(6):e0127468. doi: 10.1371/journal.pone.0127468 (PMC4466472; doi:10.1371/journal.pone.0127468)

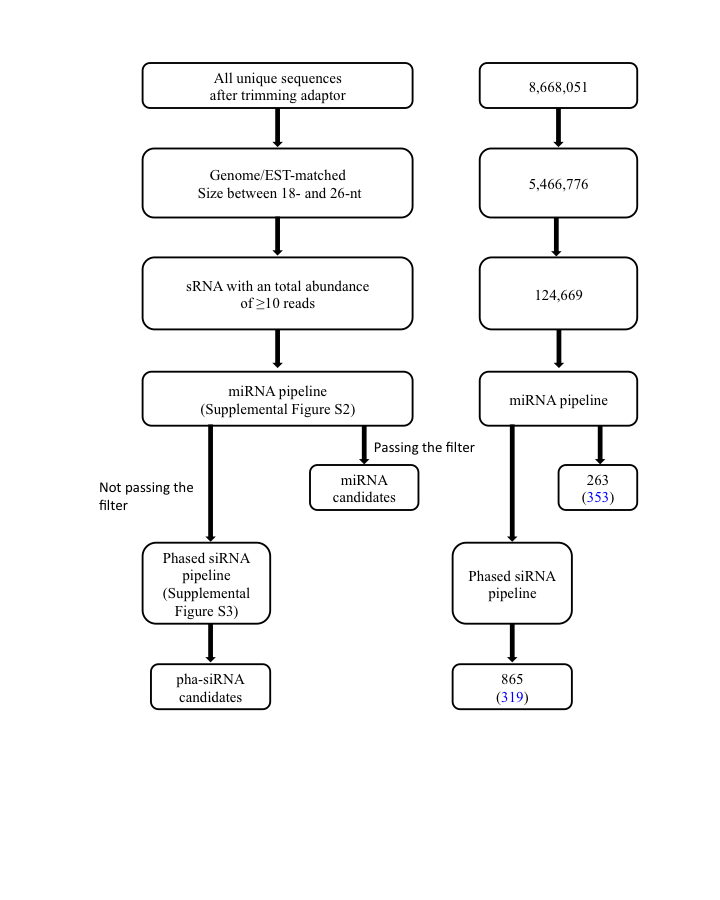

Supplement: S1 Fig — Pipeline description (shown on left) and the resulting number of sequences predicted in each stage of the pipeline (shown on right). Number of total sRNA reads (black); unique sRNA sequences (blue). (TIFF) [file pone.0127468.s001.tiff]

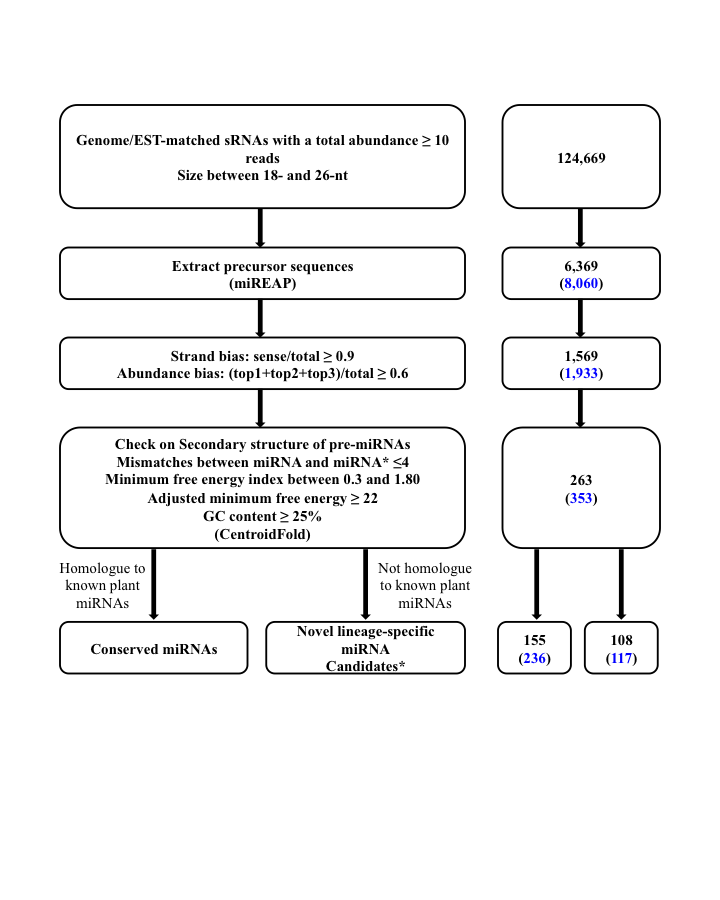

Supplement: S2 Fig — Pipleine description (shown on left) and the resulting number of sequences predicted in each stage of the pipeline (shown on right). Number of unique sRNA sequences (black); miRNA gene precursor sequences (blue). Novel lineage specific miRNA candidates were found in G. aroborem but not in other plants. (TIFF) [file pone.0127468.s002.tiff]

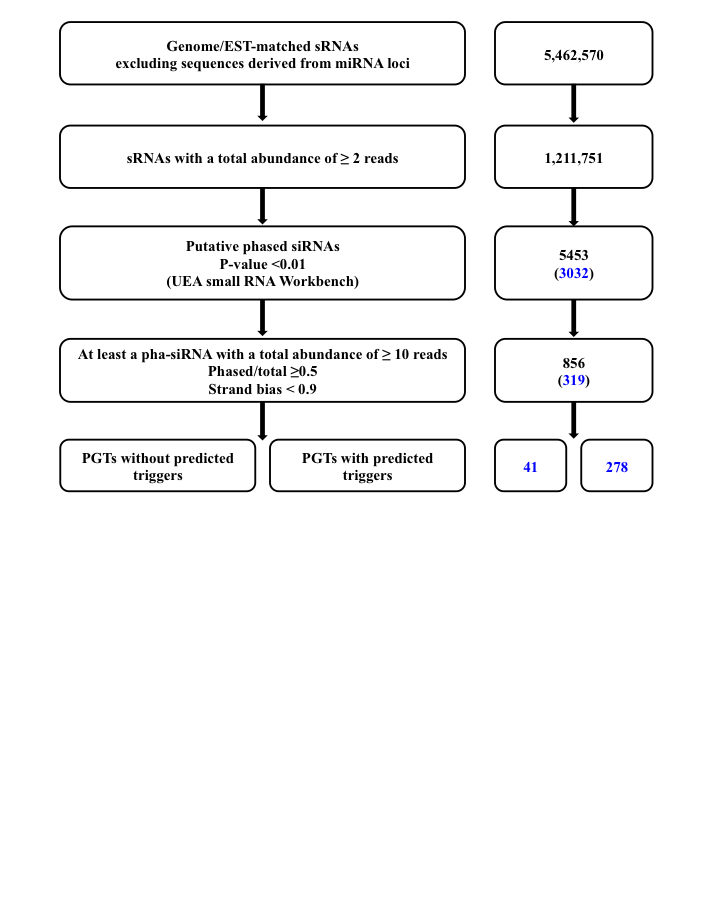

Supplement: S3 Fig — Pipeline description (shown on left) and the resulting number of sequences predicted in each stage of the pipeline (shown on right). Number of unique sRNA sequences (black); Pha-siRNA gene transcripts (blue). (TIFF) [file pone.0127468.s003.tiff]
